# Unconventional Photocatalysis in Conductive Polymers: Reversible Modulation of PEDOT:PSS Conductivity by Long‐Lived Poly(Heptazine Imide) Radicals

**DOI:** 10.1002/anie.202014314

**Published:** 2021-03-01

**Authors:** Aleksandr Savateev, Yevheniia Markushyna, Christoph M. Schüßlbauer, Tobias Ullrich, Dirk M. Guldi, Markus Antonietti

**Affiliations:** ^1^ Department of Colloid Chemistry Max Planck Institute of Colloids and Interfaces Am Mühlenberg 1 14476 Potsdam Germany; ^2^ Department of Chemistry and Pharmacy Interdisciplinary Center for Molecular Materials (ICMM) Friedrich-Alexander University Erlangen-Nürnberg Egerlandstraße 3 91058 Erlangen Germany

**Keywords:** carbon nitride, K-PHI, long-lived radicals, PEDOT, semiconductors

## Abstract

In photocatalysis, small organic molecules are converted into desired products using light responsive materials, electromagnetic radiation, and electron mediators. Substitution of low molecular weight reagents with redox active functional materials may increase the utility of photocatalysis beyond organic synthesis and environmental applications. Guided by the general principles of photocatalysis, we design hybrid nanocomposites composed of n‐type semiconducting potassium poly(heptazine imide) (K‐PHI), and p‐type conducting poly(3,4‐ethylenedioxythiophene) polystyrene sulfonate (PEDOT:PSS) as the redox active substrate. Electrical conductivity of the hybrid nanocomposite, possessing optimal K‐PHI content, is reversibly modulated combining a series of external stimuli ranging from visible light under inert conditions and to dark conditions under an O_2_ atmosphere. Using a conductive polymer as the redox active substrate allows study of the photocatalytic processes mediated by semiconducting photocatalysts through electrical conductivity measurements.

## Graphical abstract

A conductive polymer (PEDOT:PSS) and potassium poly(heptazine imide) (K‐PHI) are combined in one hybrid nanocomposite as electron donor and photocatalyst, respectively. The composite represents an example of unconventional photocatalysis: K‐PHI modulates the physicochemical properties of the conductive polymer upon exposure to light and dark in different environments.

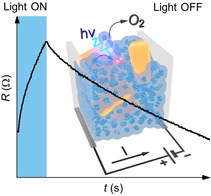

## Introduction

Semiconductor photocatalysis has been extensively studied in the area of water splitting,^[1]^ conversion of CO_2_,^[2]^ environmental applications,[^3^, ^4^] and in the synthesis of fine organic molecules.[^5^, ^6^] In all of these applications, the modus operandi involves the excitation of the semiconductor with a photon of sufficient energy followed by the quenching of the hole and electron with suitable electron donors and acceptors, respectively, in a concerted fashion.^[7]^ In simple terms, in photocatalysis, the n‐type/p‐type semiconductors regulate the flow of electrons/holes from one reagent to another without accumulating them.

In the absence of an electron acceptor, some semiconductors are able to accumulate electrons. Earlier, this feature has been confirmed for TiO_2_,[^8^, ^9^, ^10^, ^11^] ZnO,[^12^, ^13^] cyanamide‐functionalized heptazine based polymers,^[14]^ and potassium poly(heptazine imide) (K‐PHI).[^15^, ^16^] Semiconductor particles, in which electrons are accumulated near the conduction band, appear as fairly air sensitive green/blue solids and give rise to a distinct signal in the electron paramagnetic resonance (EPR) spectra.[^17^, ^18^]

Related to photocatalytic applications, electrons stored in the semiconductor reduce O_2_,[^19^, ^20^] Cr^IV^,^[10]^ methylviologen,[^12^, ^21^] octaatomic sulfur,^[15]^ phenoxyl and nitroxyl radicals.^[18]^ Long‐lived radicals of K‐PHI (hereafter referred to as (K‐PHI)^.−^) are formed upon reductive quenching of K‐PHI excited state and are key intermediates driving the synthesis of cyclopentanoles by dimerization of enones,^[22]^ dichloromethylation of enones,^[23]^ *N*‐fused pyrroles,^[24]^ or the reductive formylation of nitroarenes via multielectron transfer.^[25]^

The vast majority of known examples—including those that are aforementioned—reveal that small organic molecules and/or ions are photocatalytic reagents.[^26^, ^27^, ^28^] The employment of polymers as substrates for photocatalytic transformations has been explored significantly less.^[29]^ Prominent examples are solar reforming of biopolymers and nonrecyclable plastics.[^30^, ^31^, ^32^, ^33^] Conductive polymers are a versatile platform for designing smart materials as the electrical conductivity of the polymer follows a multitude of external physical stimuli that naturally finds application in sensing.[^34^, ^35^, ^36^, ^37^] Owing to its nontoxicity,^[38]^ flexibility, water‐solubility, processability, and commercial availability, p‐type conductive poly(3,4‐ethylenedioxythiophene) polystyrene sulfonate (PEDOT:PSS) is of great interest in the construction of functional composites and devices. In addition, 3D printing of PEDOT:PSS facilitates development of complex conductive patterns.^[39]^ Increasing the PEDOT:PSS conductivity permanently has been a topic of research for years,^[40]^ while modulation of the polymer conductivity via reversibly injecting holes (p‐doping) or electrons (n‐doping) has drawn much less attention.^[41]^ However, the basic principle of such an approach is similar to the photocatalytic redox cycle, in which reductive quenching of the photocatalyst excited state by PEDOT will result in p‐doping of the conductive polymer. In other words, PEDOT acts as an electron donor in the photocatalytic notation.

**Figure 1:**
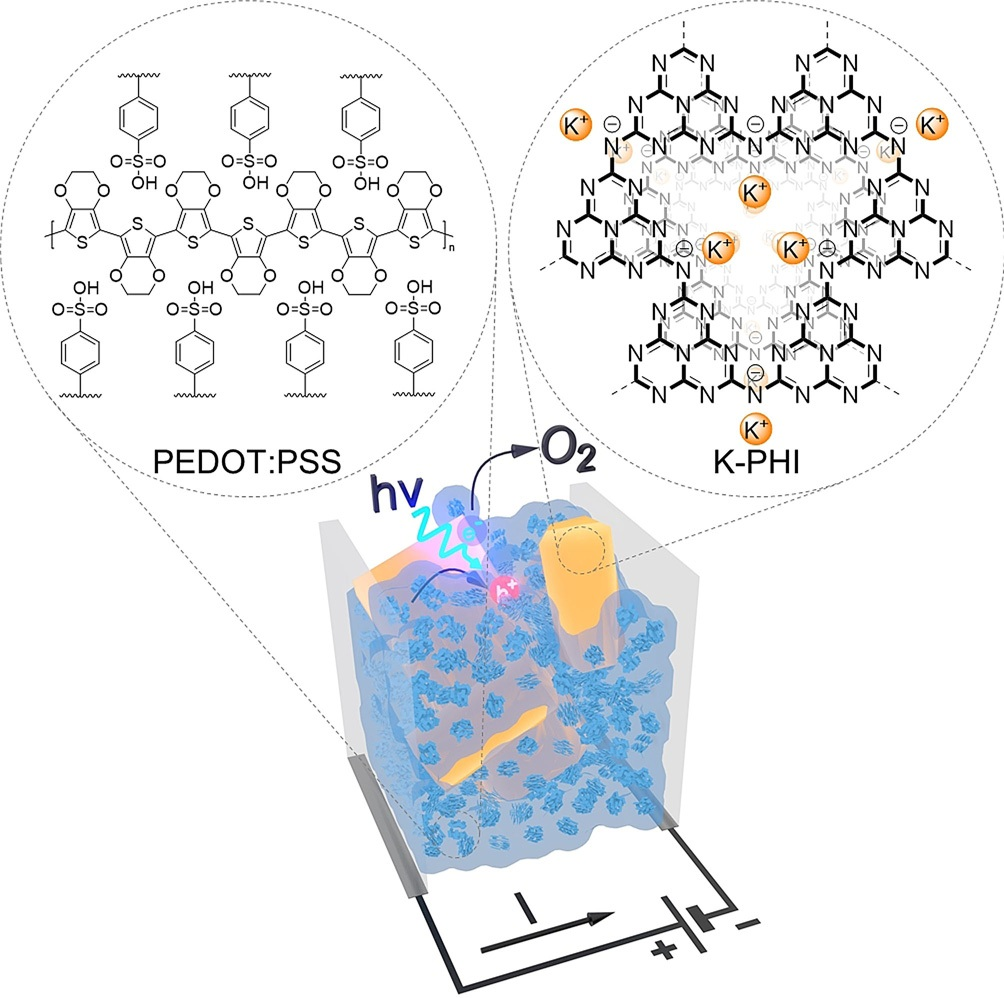
Representation of a concept to modulate the PEDOT:PSS conductivity using K‐PHI photocatalyst, O_2_ and visible light, developed in this work. Primary structures of PEDOT:PSS and K‐PHI are shown on the left and right, respectively. Key: K‐PHI nanoparticles (yellow prisms), tertiary structure of PEDOT:PSS (light blue spheres possessing a dark‐blue core).

Herein, we integrate two materials, that is, p‐type conductive polymer PEDOT:PSS, on one hand, and n‐type visible‐light responsive carbon nitride semiconductor (K‐PHI), on the other hand, into a hybrid nanocomposite: K‐PHI:PEDOT:PSS (Figure 1). Sequential exposure of K‐PHI:PEDOT:PSS to visible light under O_2_‐free conditions and to the dark under O_2_ conditions leads to the reversible doping of PEDOT:PSS that is registered as a change of the composite electrical conductivity. Only semiconductors that are capable of accumulating electrons upon light irradiation—namely to form long‐lived radicals—induce a measurable readout. We propose an unconventional photocatalytic approach. Instead of enabling a chemical reaction between small organic molecules, we use photocatalysis to tune the physicochemical properties of conductive polymers.

## Results and Discussion

**Figure 2:**
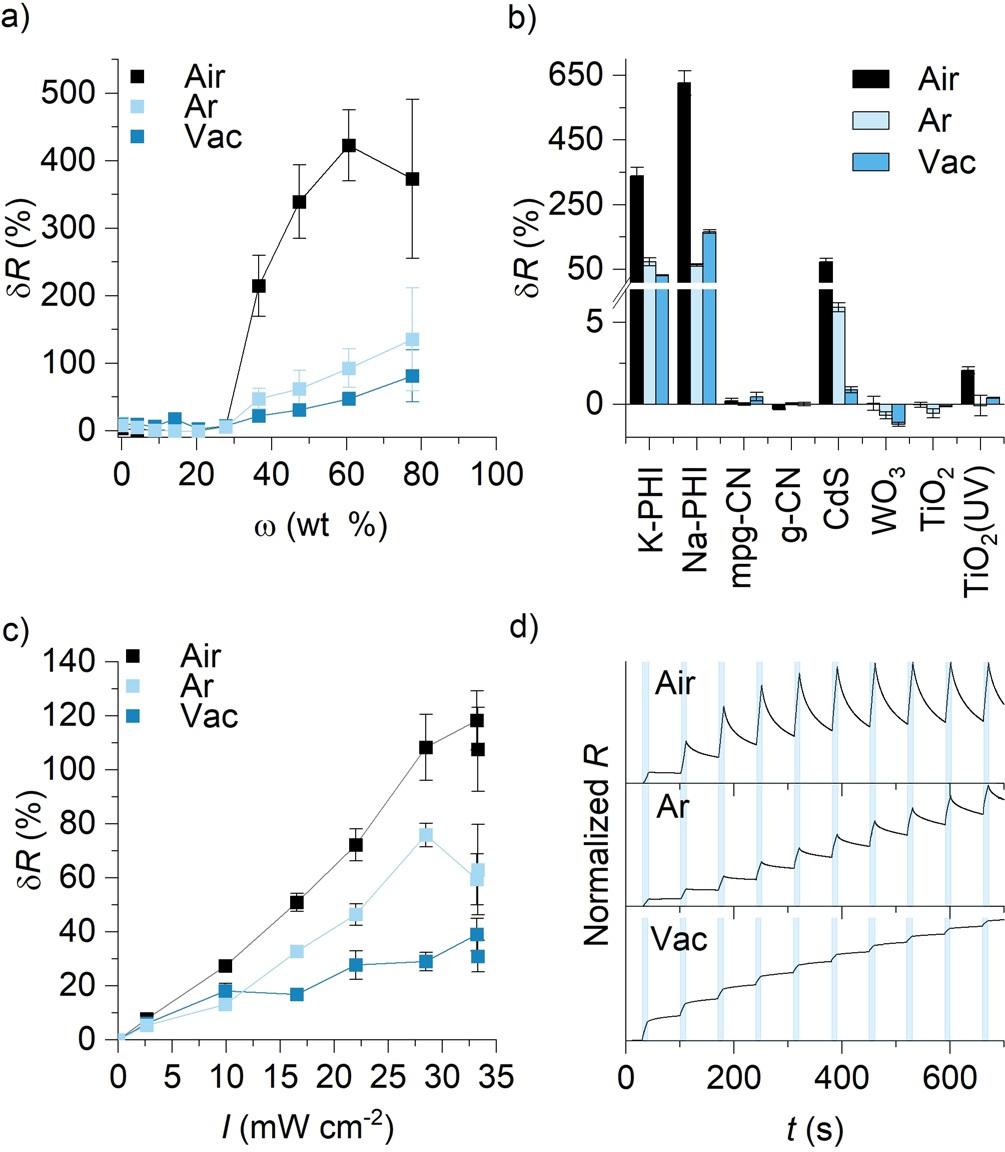
Semiconductors and screening of conditions. Error bars denote average ± std. dev. (typically three devices were prepared). Light intensity 30 mW cm^−2^ (461 nm). a) Response of the K‐PHI:PEDOT:PSS hybrid composite versus K‐PHI nanoparticle content. b) Semiconductor screening. c) Response of the composite (28 wt % K‐PHI) versus light intensity. d) Time‐dependent response of the composite (28 wt % K‐PHI) in air, Ar, and vacuum. Blue rectangles indicate the periods of light irradiation.

For high‐throughput tests a series of K‐PHI:PEDOT:PSS blends with different content of K‐PHI ranging from 0 to 77.6 wt % were prepared followed by drop casting them between two electrically isolated areas of FTO electrodes and drying in air (Supporting Information, Figures S1–S5). To check the response of K‐PHI:PEDOT:PSS hybrid nanocomposites to light, the resistance of the film was measured in the dark (*R*_D_) and after 10 s of light irradiation (*R*_L_). Consequently, the response (δ*R*) of the K‐PHI:PEDOT:PSS hybrid nanocomposite is defined as the relative resistance change under light irradiation (*R*_L_). When the measurements were performed in air, no resistance changes evolved at low K‐PHI contents, but a steep increase of response was observed at 36.5 wt % (1 bar, Figure 2 a). A percolation threshold of ca. 30 wt % K‐PHI, agrees well with the results of composite modeling made of a polymer and spherical (*d=*50 nm) Au nanoparticles.[^42^, ^43^]

In the context of our work, we rationalize the percolation threshold as follows: at concentrations of <30 wt %, K‐PHI nanoparticles are present as isolated islands that do not impact the flow of electrons. When, however, the K‐PHI content passes the 30 wt % threshold, long‐range order of K‐PHIs and, in turn, connectivity between them is realized. At a K‐PHI content of ≥77.6 wt %, a random close packing (RCP) is reached that results in a nonconductive composite. Higher RCPs than for ideal spheres (ca. 64 %) are due to the non‐uniform (60–200 nm) diameters of K‐PHI particles.^[23]^

To read out the response of the K‐PHI:PEDOT:PSS nanocomposites, hereafter we report data using a K‐PHI content of 28 wt %. Notable, exposure of the hybrid nanocomposites to light in vacuum (0.1 mbar) or under Ar (1 bar) (Figure 2 a) evokes much weaker changes.

Using a small scope of carbon nitride based materials, we confirmed that only long‐lived radical forming^[21]^ K‐PHI and Na‐PHI showed significant responses of 354 % and 635 %, respectively, in air (Figure 2 b). We opted for K‐PHI despite its moderate response due to the smaller diameter and, in turn, better dispersibility to prepare more homogeneous films.

Among inorganic semiconductors, CdS gave rise to a sizeable response under visible‐light irradiation: 73±11 % in air. TiO_2_, in contrast, lacked any response at all under blue light irradiation, but showed weak response under 365 nm irradiation: 2±0.2 % in air. Significantly better responses of the composites based on K‐PHI and Na‐PHI relative to CdS and TiO_2_ signify a better interaction of the carbon nitride materials with PEDOT, presumably due to the π‐conjugated structures present in both carbon nitrides and the conductive polymer.

The dependence of the K‐PHI:PEDOT:PSS nanocomposite response on the light intensity is nearly linear regardless of the environment, that is, air, Ar, or vacuum (Figure 2 c). Already under dim light of 2.6 mW cm^−2^ at 461 nm we recorded a 8 % response. Some examples of the normalized resistance versus time in air alternating dark (60 s) and light (10 s) cycles are summarized in Figure 2 d.

Data analyses indicate that the nanocomposite shows a steep near‐linear resistance change under light irradiation followed by a slow recovery in the dark. The latter is best described by a one‐exponential fitting function (R^2^=0.997, Figures S6 and S7). Therefore, formation of (K‐PHI)^.−^ in the light cycle is much faster than its quenching with O_2_ in the dark cycle.^[44]^ Quenching is a first‐order reaction with a distinct concentration dependence (see below). Time‐dependent experiments under argon showed a steep increase of the electric resistance in the light cycle followed by only partial recovery in the dark cycle due to trace amount of O_2_ (Figure 2 d). In vacuum with a maximum concentration of residual O_2_ of ≤20 ppm, recovery of conductivity in the dark cycle is negligible.

In the three cases, which are shown in Figure 2 d, the baseline varies. This suggests that (K‐PHI)^.−^, presumably those located deep in the bulk of the nanocomposite, are incompletely quenched during the dark cycle. In parallel testing with three films, a relative standard deviation of <5 % in air and Ar as well as of <10 % in vacuum was determined (Figure S8). Control measurements at different temperatures ruled out any composite heating to cause the resistance change (Figure S9). I‐V curves of the composite confirm the stability in the range between −5 and +5 V (Figure S10).

**Figure 3:**
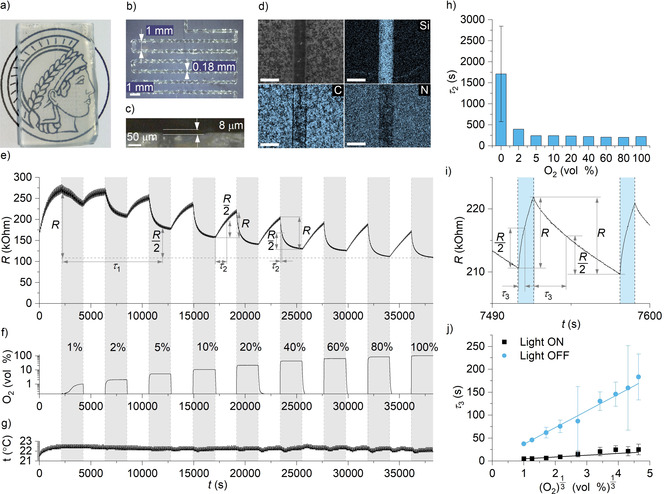
Tests of the K‐PHI:PEDOT:PSS film at the patterned FTO electrode. a) Film deposited by spray coating at the patterned electrode. b,c) Optical microscope images of the device. d) SEM and Si, C and N EDX maps. e) Time‐dependent electric resistance (R) of the K‐PHI:PEDOT:PSS film. Derived half‐life values τ_1_ and τ_2_ (longer in an O_2_‐free environment and shorter in the presence of O_2_) are shown. Grey rectangles indicate the period of time when the electrode was exposed to O_2_. f) Time‐dependent O_2_ concentration in the chamber (semi logarithmic scale). g) Gas temperature in the chamber. h) Half‐life of the composite resistance changes at different O_2_ concentrations. For an O_2_‐free environment the mean and std. dev. (*n*=9) are reported. i) Explanation of the half‐life τ_3_ parameter, where τ_3_ is shorter under light irradiation and longer in the dark. Blue rectangles indicate the periods of light irradiation. j) Half‐life τ_3_ versus ^3^ $\sqrt{C}$ shows linear dependence. Data points represent the mean and std. dev. (*n*=30).

Next, we investigated the influence of O_2_ concentration in the gas carrier on the kinetics of how K‐PHI interacts with PEDOT:PSS by “inverting the sensing Scheme” under constant light illumination using patterned FTO electrodes. K‐PHI:PEDOT:PSS films were deposited on FTO electrodes by means of spray coating (Figure 3 a–d; Figures S11–23). Throughout the experiments the temperature remained constant at 22±1 °C. The R versus time dependence is rather complex. The global baseline was fit by a one‐exponential decay function featuring a half‐life τ_1_ of 1.2×10^4^ s suggesting gradual p‐doping of PEDOT (Figure 3 e).

Each of the data set of up to 30 cycles under defined O_2_ concentration (Figure 3 f) and temperature (Figure 3 g) was fit with a one‐exponential fitting function to afford a half‐life τ_2_. Under O_2_‐free conditions the value is (1.7±1.1)×10^3^ s (Figure 3 h). In the presence of O_2_ (2–100 vol %), the half‐life is shorter, 200–400 s, and depends weakly on O_2_ concentration.

Finally, each of the 540 cycles of the R built‐up upon irradiation with light as well as the R decrease in the dark was fit with one‐exponential fitting functions (Figure 3 i). The half‐life (τ_3_) of both processes depends linearly on ^3^ $\sqrt{C}$ (Figure 3 j). However, the dependence is more pronounced for the dark cycle with a slope of 36 s [vol %]^3^, compared to the light cycle with a slope of 4 s [vol %]^3^. In short, despite the fact that several processes occur in the nanocomposites they differ in their characteristic lifetimes by one order of magnitude. Hereby, the fastest process is triggered by irradiation with light. Although the morphology and the preparation both affect the film conductivity,^[45]^ our data corroborates that regardless of the film preparation technique the hybrid nanocomposites give rise to the same response to an external stimuli.

### Spectroscopic studies

#### Electron paramagnetic resonance (EPR) spectroscopy

**Figure 4:**
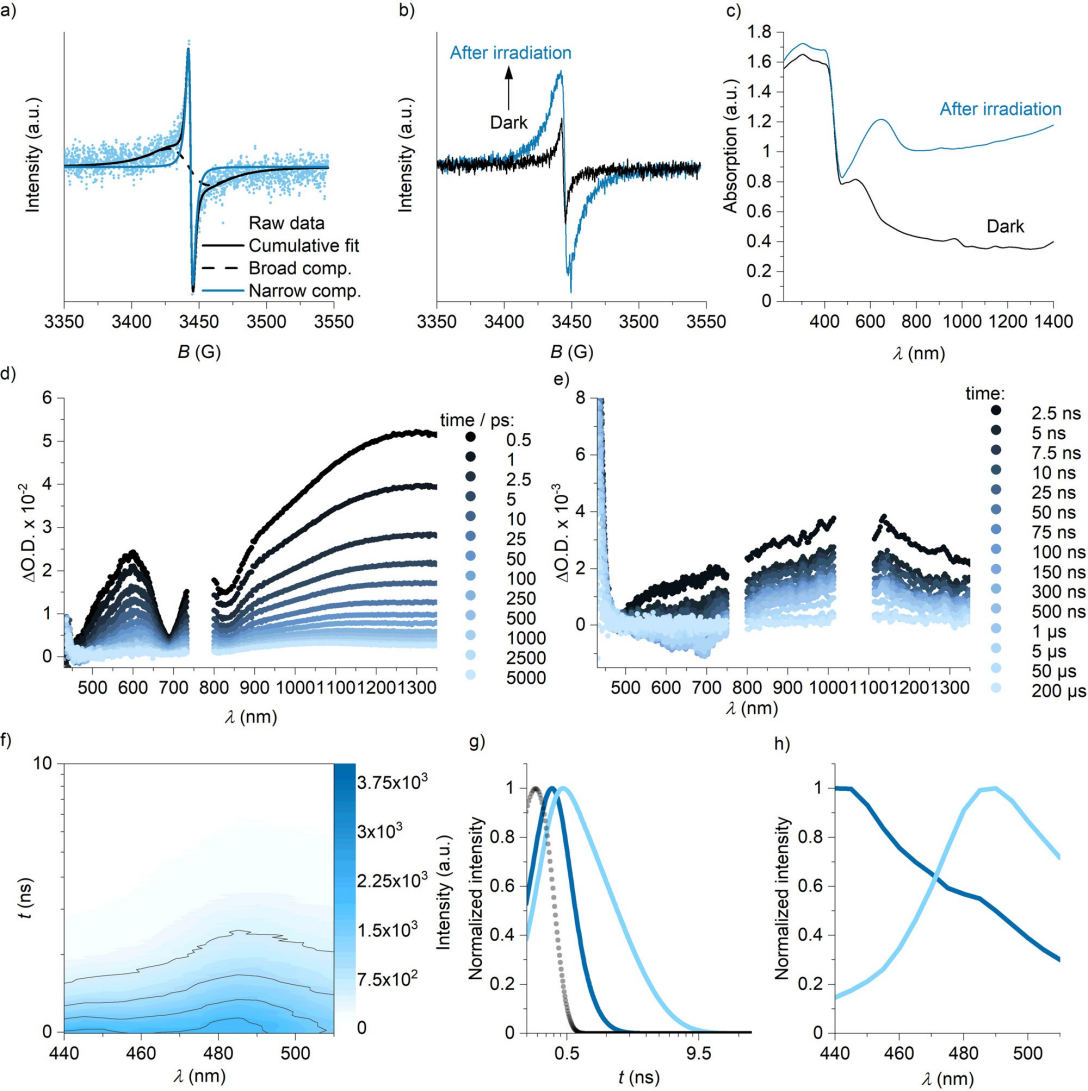
Spectroscopic studies. a) EPR spectrum K‐PHI:PEDOT:PSS acquired at 300 K. Narrow component, broad component and cumulative fit are shown. b) EPR spectra of K‐PHI:PEDOT:PSS acquired in the dark, immediately upon irradiation with light, and after relaxation for a specified period of time in the dark. c) DRUV‐vis absorption spectra of K‐PHI^.−^ as a suspension in benzylamine and reference K‐PHI. d) Transient absorption spectra of K‐PHI:PEDOT:PSS film on a glass slide acquired in the ps–ns range. e) TAS of K‐PHI:PEDOT:PSS film on a glass slide acquired in the ns–μs range. λ_exc_=387 nm. f) Plot of time vs. photoluminescence wavelength for K‐PHI:PEDOT:PSS. g) Deconvolution of the TRES data with GloTarAn (global analysis) with two species, their individual time evolution and modeled instrument response function (IRF, block dots). h) The deconvolution of the TRES data of the two species taken into account for global analysis. Deconvoluted spectra of species 1 (dark blue) and species 2 (light blue).

To gain insights into the mechanism of modulating the film conductivity using light and O_2_, we started our investigation with K‐PHI, PEDOT:PSS, and K‐PHI:PEDOT:PSS in the solid state by means of variable‐temperature EPR spectroscopy (Figures S24–S29).^[46]^ EPR signals of PEDOT:PSS as well as that of K‐PHI:PEDOT:PSS are well fit by two Lorentzian derivatives with g‐factors of 2.003. Implicit, is the presence of localized polarons, that is, the narrow component, and delocalized polarons, that is, the broad component, in PEDOT:PSS (Figure 4 a).^[47]^ Polarons hereafter are referred to as (PEDOT)^.+^. Studying the materials by EPR spectroscopy at room temperature and under irradiation with light (Figure 4 b; Figures S30–S34) revealed that the K‐PHIs exert a clear impact on the charge carrier mobility in PEDOT:PSS. On one hand, irradiation of the PEDOT‐based materials with light triggers the conversion of bipolarons (PEDOT)^2+^, which are two orders of magnitude more mobile, into (PEDOT)^.+^.[^48^, ^49^] On the other hand, under light irradiation, K‐PHI “catalyzes” the conversion of (PEDOT)^2+^ into (PEDOT)^.+^ compared to just PEDOT:PSS. For example, for K‐PHI:PEDOT:PSS the concentration of polarons increases ca. 3 times, while for PEDOT:PSS only by ca. 5 % (Figure S32). Studying the kinetics of polaron decays revealed its reversibility (Figure S32).^[50]^ All together, we explain the decrease of PEDOT:PSS conductivity by a decrease of (PEDOT)^.+^ mobility in the presence of K‐PHI triggered by light irradiation.

To determine the lifetime of the corresponding steps in this photocatalytic process and to correlate them with τ_2_ and τ_3_ from the electric measurements, we conducted spectroscopic studies using absorption and emission steady‐state and time‐resolved techniques.

### Steady‐state absorption

K‐PHI films show intense absorptions below 460 nm that tails into the near IR (nIR) region of the optical spectrum, which corresponds to low‐energy intraband transitions (Figure S35). For (K‐PHI)^.−^, we find absorptions below 460 nm similar to that of the K‐PHI ground state and maximum at around 660 nm followed by broad absorptions between 750 nm and the nIR region (Figure 4 c; Figure S36).^[21]^ For PEDOT:PSS, a broad absorption of low intensity is discernable at around 850 nm, which is commonly assigned to (PEDOT)^.+^, and a much broader and more intense absorption >1400 nm, which is due to (PEDOT)^2+^ (Figure S35).^[51]^ The addition of K‐PHIs to PEDOT:PSS leads to partial deintensification of the (PEDOT)^2+^ fingerprint and to an intensification of that of the (PEDOT)^.+^ (Figure S35).^[51]^

### Transient absorption spectroscopy

To gather insights into the interactions of K‐PHI with PEDOT:PSS upon irradiation with light we compared pristine films of K‐PHI (Figure S37) and PEDOT:PSS (Figure S38) with those of KPHI:PEDOT:PSS (Figure 4 d,e) by means of transient absorption spectroscopy (TAS) with sub‐picosecond and nanosecond temporal resolutions.^[52]^ Directly after 387 nm photoexcitation of the K‐PHI:PEDOT:PSS nanocomposite ground state bleaching (GSB) develops at around 440 nm together with excited state absorption (ESA) at 600, 760, and >900 nm. A virtual minimum at 690 nm is also discernible due to the overlap of (K‐PHI)^.−^ GSB and ESA (Figure 4 d). In contrast to pristine K‐PHI, the ESA at >900 nm maximizes at around 1300 nm, which is in line with the (PEDOT)^2+^ fingerprint seen in the reference measurements with PEDOT:PSS (Figures S35 and S38). Within 2 ns, these positive differential absorptions transform into a broad and long‐lived 1050 nm maximum. Its decay is, however, masked by the instrumental time resolution. Similar to the control experiments with K‐PHI (Figure S37), we noted that the 600 nm maximum red‐shifts to 615 nm within the first nanoseconds together with the formation of a long‐lived 440 nm feature. Experiments on the μs‐timescale reveal that the ESA ranging from 500 to 750 nm quickly transforms into a 700 nm GSB. As a matter of fact, this is strongly reminiscent to the GSB found in reference experiments with PEDOT:PSS (Figure S38). Thus, we assign the negative signal to the depopulation of the neutral state within the PEDOT chains. The initial, short‐lived ESA at 1300 nm is assigned to a combination of ESA stemming from K‐PHI and (PEDOT)^2+^, whereas the long‐lived ESA 1050 nm species is assigned to (PEDOT)^.+^. All of our findings prompt to the fact that upon photoexcitation electrons are transferred from (PEDOT)^0^ to K‐PHI leading to the formation of the (PEDOT)^.+^ (K‐PHI)^.−^ radical ion pair. In other words, upon photoexcitation K‐PHI oxidizes PEDOT. Back electron transfer that is expected to lead to the recovery of K‐PHI and (PEDOT)^0^ is suppressed due to stability of the formed pair.^[53]^

### Time‐resolved emission spectroscopy

Steady state photoluminescence (PL) spectrum of K‐PHI:PEDOT:PSS is composed of peaks of individual materials, while intensity is two times lower compared to K‐PHI, suggesting that radiative recombination of excitons is suppressed due to formation of (PEDOT)^.+^ (K‐PHI)^.−^ radical ion pair (Figure S39). To complement the steady‐state photoluminescence intensity decrease, we investigated the radiative recombination of excitons by time‐resolved emission spectroscopy (TRES) upon 365 nm photoexcitation by detecting the PL between 440 and 530 nm (Figure 4 f–h). Two radiative species were found with PL maxima at 440 and 490 nm in the cases of K‐PHI (Figure S40) and K‐PHI:PEDOT:PSS (Figure 4 h). For K‐PHI, these decay with lifetimes of 0.4 and 2.5 ns (Figure S40b), while for K‐PHI:PEDOT:PSS, the latter is slightly quenched to 2.0 ns (Figure 4 g), corroborating quenching of radiative recombination of excitons due to the formation of stable (PEDOT)^.+^ (K‐PHI)^.−^ radical ion pairs, as already indicated by steady‐state experiments.

### Mechanism

**Figure 5:**
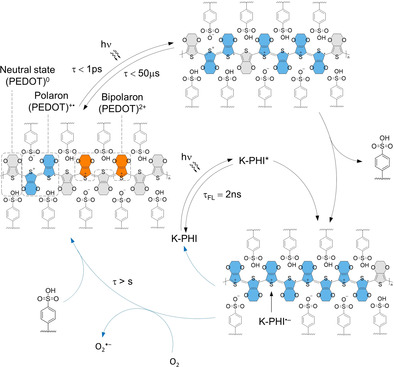
A photocatalytic mechanism. The corresponding lifetimes of the processes derived from the spectroscopic study are shown. Color code for the fragments of PEDOT:PSS are the following: (PEDOT)^0^ (grey), (PEDOT) ^.+^ (blue), (PEDOT)^2+^ (orange).

Based on our spectroscopic data, we propose the following photocatalytic mechanism that explains the modulation of PEDOT:PSS electrical conductivity in the presence of K‐PHI triggered by light (Figure 5). In the first step, irradiation of PEDOT:PSS with blue light triggers a 1 ps lasting electron transfer from neutral (PEDOT) to (PEDOT)^2+^ to create two (PEDOT)^.+^. In the absence of K‐PHI nanoparticles, the recovery of the ground state occurs within 50 μs. At the same time, absorption of blue photons by K‐PHI leads to the formation of the K‐PHI excited state (K‐PHI)*. Taking into account the more positive position of the valence band in K‐PHI, +2.13 V vs. NHE,^[15]^ compared to (PEDOT)^0^, +0.92 V,^[54]^ reductive quenching of K‐PHI* leads to the formation of (K‐PHI)^.−^ and increases further population of (PEDOT)^.+^. Once formed, the negative charges of (K‐PHI)^.−^ nanoparticle are compensated by positively charged (PEDOT)^.+^ that together form a stable radical ion pair.^[55]^ Stability of (PEDOT)^.+^ (K‐PHI)^.−^ under anaerobic conditions is rationalized on the following terms. Accumulation of electrons in K‐PHI nanoparticles shifts the zeta‐potential to more negative values (Figure S41a). Due to electrostatic attraction, (K‐PHI)^.−^ interacts stronger with PEDOT compared to PSS (Supporting Information, Table S4). In Figure 5, this process is schematically depicted as substitution of one benzenesulfonic moiety with (K‐PHI)^.−^. Although the PSS matrix plays an essential role in electron transport,^[56]^ (K‐PHI)^.−^ strongly “binds” (PEDOT)^.+^ as well as (PEDOT)^2+^ and eventually decreases their mobility. Importantly, the conductivity of PEDOT‐based materials depends on the structure of the polyelectrolyte and is, in general, the highest for polystyrene derivatives.[^57^, ^58^] Using a variety of polyelectrolytes, Hadziioannou and Cloutet have shown that the conductivity of the PEDOT:polyelectrolyte increases with the acidity of the polyelectrolyte.^[59]^ From this perspective, in situ partial substitution of acidic PSS with basic (K‐PHI)^.−^,^[60]^ is expected to decreases the conductivity of the hybrid composite. In‐depth analyses by means of Fourier Transform Infrared (FT‐IR) spectroscopy and measurements of surface zeta‐potential were conducted. They suggest that the ionic structure of K‐PHI due to the negatively charged polymeric scaffold and the potassium counter ions is crucial. It enables binding to PEDOT:PSS, while the process can be regarded as ion exchange reaction (Figure S41b–d).

Stopping the light irradiation and the exposure to O_2_ quenches (K‐PHI)^.−^ and enables the recovery of the conductivity in the hybrid nanocomposite. Quenching of (K‐PHI)^.−^ occurs on the time‐scale exceeding seconds (τ> s), which is much slower compared to the formation of (K‐PHI)^.−^ as confirmed by electrical conductivity measurements, EPR, TAS and earlier reports.[^14^, ^15^, ^44^] Interactions with O_2_ via electron transfer and subsequent formation of superoxide radical has been proposed in numerous photocatalytic reactions involving carbon nitrides,^[19]^ energy transfer with the formation of singlet oxygen is yet another alternative.[^61^, ^62^]

Electrochemical p‐doping of polythiophene‐based materials is typically performed at potentials of around +1 V.[^63^, ^64^] Potentials higher than +1.4 V might lead to the overoxidation of the polymer, albeit reversible.[^65^, ^66^] We found that the hybrid composite material retains its responsivity to external stimuli for several hundred cycles (Figure 3 e). Therefore, in this work, PEDOT serves as a redox active material that participates in (quasi)reversible electron transfer.

## Conclusion

Hybrid nanomaterials composed of K‐PHI nanoparticles and the conductive PEDOT:PSS matrix were prepared by a straightforward method. The electrical conductivity of K‐PHI:PEDOT:PSS shows a response to light irradiation at K‐PHI content of ca. 30 wt %, which is rationalized by the formation of long‐range connectivity channels between (K‐PHI)^.−^ upon photocharging. Overall, the electrical conductivity was correlated well with the results of EPR, steady‐state and transient absorption spectroscopy. The results strongly suggest that the processes in the hybrid nanocomposite, which were triggered by light, are best described by what is commonly used in organic photoredox catalysis. In particular, the K‐PHI excited state undergoes reductive quenching by PEDOT to afford (K‐PHI)^.−^ and p‐doping of PEDOT. Important is that accumulation of electrons near the conduction band has several effects. It increases the zeta‐potential of K‐PHI nanoparticles, facilitates the localization of (PEDOT)^.+^, decreases the mobility, and decreases the electrical conductivity. In the photocatalyst turnover step, upon exposing the hybrid nanocomposite to an electron acceptor, such as O_2_, (K‐PHI)^.−^ is re‐oxidized. As a result, the (PEDOT)^.+^ mobility in PEDOT:PSS is gradually recovered and the electrical conductivity is restored.

## Conflict of interest

A patent WO/2019/081036 has been filed by Max Planck Gesellschaft zur Förderung der Wissenschaften E.V. in which A.S. and M.A. are listed as a co‐authors.

## Supporting information

As a service to our authors and readers, this journal provides supporting information supplied by the authors. Such materials are peer reviewed and may be re‐organized for online delivery, but are not copy‐edited or typeset. Technical support issues arising from supporting information (other than missing files) should be addressed to the authors.

Supplementary
