# Supplementary material for: Unconventional Photocatalysis in Conductive Polymers: Reversible Modulation of PEDOT:PSS Conductivity by Long‐Lived Poly(Heptazine Imide) Radicals
Source: Angew Chem Int Ed Engl. 2021 Mar 1;60(13):7436–43. doi: 10.1002/anie.202014314 (PMC8048452; doi:10.1002/anie.202014314)
Supplement: Supplementary file 1 — Supplementary [file ANIE-60-7436-s001.pdf]

Angewandte Chemie
Eine Zeitschrift der Gesellschaft Deutscher Chemiker

Supporting Information

Unconventional Photocatalysis in Conductive Polymers: Reversible Modulation of PEDOT:PSS Conductivity by Long-Lived Poly(Heptazine Imide) Radicals

Aleksandr Savateev[+], Yevheniia Markushyna[+], Christoph M. Schüßlbauer, Tobias Ullrich, Dirk M. Guldi, and Markus Antonietti*

anie_202014314_sm_miscellaneous_information.pdf

Supporting Information

‡

Contents

1. Materials...2

2. Equipment 3

3. Methods 5

3.1. Steady-state fluorescence spectroscopy 5

3.2. Fourier transform infrared (FT-IR) spectroscopy 5

3.3. Scanning electron microscopy (SEM) and energy dispersive X-Ray (EDX) analysis 5

3.4. Steady state absorption spectroscopy 5

3.5. X-Ray diffraction 5

3.6. EPR study 5

3.7. AFM study..6

3.8. Time resolved emission spectry (TRES) / time correlated single photon counting (TCSPC) 6

3.9. Ultrafast pump-probe transient spectroscopy evaluation...6

3.10. Elemental analysis...7

3.11. Zeta-potential measurements...7

3.12. Photocatalytic oxidation of PEDOT:PSS by K-PHI in water 7

4. Synthesis of carbon nitrides...8

5. Fabrication of FTO electrodes, spray coating of the electrodes with the materials, study of response to different stimuli and data processing 10
5.1. Preparation of K-PHI:PEDOT:PSS mixture 10
5.2. Type A FTO electrode. 10
5.3. Type B FTO electrode. 11
6. Supplementary discussion 14
6.1. Steady-state fluorescence 14
6.2. EPR study at variable temperature 14
6.3. EPR study under light irradiation. 15
6.4. AFM study 16
6.5. TAS 17
6.6. Photocatalytic oxidation of PEDOT:PSS by K-PHI in aqueous medium 18
7. Supplementary Figures 19
8. Supplementary Tables 49
9. References 52

1. Materials

5-aminotetrazole monohydrate (>98%) was purchased from TCI; LiCl was purchased from Fisher Scientific; KCl (≥ 99.5%), NaCl (≥99.8%) were purchased from Carl Roth; Melamine (99%), Ludox® HS-40 (40 wt. % dispersion), PEDOT:PSS (1.3 wt. % dispersion in water; PEDOT content 0.5 wt.%, PSS content 0.8 wt.%), CdS (98+%), TiO_2 (99.5%), WO_3 (99.9%) were purchased from Sigma-Aldrich; Dicyandiamide (99%) was purchased from Alfa Aesar; Ar 5.0 (99.999%, H_2O ≤3 vol. ppm), Nitrogen 5.0 (99.999%, H2O ≤3 vol. ppm, O_2≤2 vol. ppm), Oxygen 5.0 (99.999 vol.%, H_2O 3 vol. ppm) were purchased from Westfalen. Reagents were used as received without additional purification.

2. Equipment

CO_2-laser

Electrodes were fabricated by engraving FTO-coated glass slides with a benchtop laser engraver (Trotec Speedy 100 equipped with a 60 W CO_2-laser). For the engraving process the laser was operated in pulsed mode with a pulse frequency of 1000 Hz and an effective power of 0.4 W.

Laboratory power supply

Manson HCS 3202 was used as a source of constant voltage.

Multimeter

Measurement of electric resistance and current were performed using digital multimeter RSDM3055. Data was recorded and stored on the computer using LabView based software developed at the electric workshop of the institute.

O_2 and temperature sensor

O_2 concentration (% O_2) and temperature (°C) were measured and logged using Presens Fibox 3. PSt3 (detection limit 15 ppb) was used as O_2 sensor. Prior data logging, the oxygen sensor was calibrated by two points: 1) N_2 (100%) and 2) air (Elevation above sea level: 34 m). The data was dynamically averaged by 4 points. Sampling rate was 1 data point per second. Data was recorded and stored on the computer using OxyView V6.02 software provided by Presens.

Mass flow controllers

Mass flow controllers (MFCs) have been used to prepared gas mixtures of desired composition. Bronkhorst MFC calibrated for N_2 was used to provide steady flow of N_2. Bronkhorst MFC calibrated for NH_3 was used to provide steady flow of O_2. The difference between O_2 and NH_3 physical properties has been taken into account using database provided by the MFCs (https://www.fluidat.com/).

Light source

LED with emission maximum 461 nm and optical power 30mW cm^2. The LED module was connected to the PC via home-made adaptor to program light ON/OFF cycles. The adaptor was controlled via LabView based software developed at the electric workshop of the institute.

Light intensity of the LED module was measured using PM400 Optical Power and Energy Meter equipped with the integrating sphere S142C and purchased from Thorlabs. Emission spectrum is shown in Figure S41.

Air brush

Fengda Model: BD-186, cup capacity 5 mL, nozzle diameter 0.3 mm. Air brush was connected to compressed air (2 bar).

3. Methods

For spectroscopic studies, glass slide or FTO glass was spray coated with K-PHI, PEDOT:PSS or K-PHI:PEDOT:PSS unless other method for samples preparation is noted.

3.1. Steady-state fluorescence spectroscopy

Fluorescence spectra were recorded on Jasco FP-8300 fluorescence spectrometer. The excitation wavelength was set to 360 nm.

3.2. Fourier transform infrared (FT-IR) spectroscopy

Spectra were recorded on Thermo Scientific Nicolet iD5 spectrometer.

3.3. Scanning electron microscopy (SEM) and energy dispersive X-Ray (EDX) analysis

Analysis has been conducted on a LEO 1550-Gemini microscope equipped with Oxford Instruments EDX detector.

3.4. Steady state absorption spectroscopy

Absorption spectra were acquired using Shimadzu UV 2600 in transmission mode.

K-PHI long-lived radical has been prepared by mixing K-PHI (20 mg) and benzylamine (20 µL) between two quartz glass slides and placed into the sample holder followed by irradiation with blue LED for 30 s. Absorption spectra of green K-PHI long-lived radical and K-PHI suspension prior irradiation with light (a reference) were acquired in diffuse reflectance mode.

3.5. X-Ray diffraction

X-Ray diffraction patterns were recorded on Bruker D8 Advance diffractometer equipped with a scintillation counter detector with CuKα radiation (λ = 0.15418 nm) applying 2θ step size of 0.05° and counting time of 3s per step.

3.6. EPR study

EPR study was conducted using Bruker EMXnano benchtop X-Band EPR spectrometer. A capillary sealed from one end was charged with the corresponding material:

K-PHI. A suspension of K-PHI nanoparticles in water (2.5 mL, 5 mg mL^{-1}) was evaporated till dryness at 20-25°C and atmospheric pressure. The mass of K-PHI taken for the analysis 6.1 mg.

PEDOT:PSS. A solution of PEDOT:PSS (2.5 mL, 1.3 wt. %) was evaporated till dryness at 20-25°C and atmospheric pressure followed by grinding. The mass of PEDOT:PSS taken for analysis 6.0 mg.

K-PHI:PEDOT:PSS (K-PHI 28 wt. %). A mixture consisting of K-PHI nanoparticles in water (2.5 mL, 5 mg mL^{-1}) and PEDOT:PSS in water (2.5 mL, 1.3 wt. %) was evaporated till dryness at 20-25°C and

atmospheric pressure followed by grinding. The mass of K-PHI:PEDOT:PSS taken for analysis 5.9 mg.

The capillary was placed into an EPR tube (ID 3 mm, OD 4 mm, length 250 mm). The following settings have been used for spectra acquisition at room temperature: Centre Field 3445.65 G, Sweep Width 200 G, Receiver Gain 40 dB, Modulation Amplitude 1.000 G, Number of Scans 1, Microwave Attenuation 25 dB (equivalent to power 0.3162 mW). For acquisition of EPR spectra at 300K, 280K, 260K, 240K, 220K, 200K, 180K, 160K, 140K, 120K, 100K and 90K microwave attenuation was set to 33 dB. Uncertainty of temperature setting was ±1K. Three samples have been analysed in parallel. To study influence of light irradiation on paramagnetic centres in K-PHI, PEDOT:PSS and K-PHI:PEDOT:PSS, capillaries filled with the materials have been evacuated to the residual pressure 7 mbar and refilled with argon. EPR spectra of the materials treated in this way have been acquired. The samples were irradiated with blue light for 5 min followed by EPR spectra acquisition. Finally, capillaries have been evacuated to the residual pressure 7 mbar and refilled with air. A series of EPR spectra was acquired after ca. 1Fitting of the EPR spectra was performed using tools in Xenon nano software (Bruker). Standard deviation of specific concentration of radicals in K-PHI and polarons in PEDOT:PSS and K-PHI:PEDOT:PSS, line width and signal amplitude were calculated based on three measurements performed for each sample in parallel under identical conditions.

3.7. AFM study

Sample was deposited at mica substrate using spray-coating. Analysis has been conducted on Veeco Dimension 3100 atomic force microscope.

3.8. Time resolved emission spectry (TRES) / time correlated single photon counting (TCSPC)

TRES/TCSPC experiments were recorded with a FS5-TCSPC spectrofluorometer from Edinburgh Instruments equipped with a photomultiplier R928P emission detector. Samples were excited by a VISUV versatile picosecond laser module from Picoquant. The repetition rate was 8 MHz. TRES data were evaluated using the TIMP based GloTarAn program.[1] The instrument response function (IRF) was modelled and taken into account. The same samples as for transient absorption experiments were used. All experiments were conducted under air.

3.9. Ultrafast pump-probe transient spectroscopy evaluation

Ultrafast pump-probe transient absorption spectroscopy (TAS) was performed using a Clark MXR CPA 2101 Ti:sapphire as the laser source (775 nm, 1 kHz, 150 fs pulse width). To acquire the time-resolved transient absorption spectra on a sub-ps and ns resolution, an Ultrafast Systems HELIOS or EOS fs/ns transient absorption spectrometer was used with time delays from 0 to 5500 ps and 1 ns to 400 µs, respectively. For sub-ps, white light for the probing pulse in the visible region of

the optical spectrum (~420-770 nm) was generated by focusing part of the fundamental 775 nm output onto a 2 mm sapphire disk. For (near) IR (800-1350 nm) white light, a 10 mm sapphire was used. For ns timescale experiments, white light for probing was generated by a photonic crystal fiber supercontiuum laser with a 1064 nm fundamental. The excitation wavelength was generated via the second harmonic of the fundamental CPA laser wavelength and the energy per pulse reduced to 2 µJ using neutral density filter.

3.10. Elemental analysis

Combustion elemental analysis has been performed on Vario Micro device. PEDOT:PSS and K-PHI:PEDOT:PSS powders for elemental analysis were obtained by evaporation of water under reduced pressure (100 mbar).

3.11. Zeta-potential measurements

Measurements were performed in water using Malvern Zetasizer.

To monitor change of K-PHI nanoparticles zeta-potential upon photocharging, a suspension of K-PHI nanoparticles (0.5 mg mL^{-1}) in benzylamine (1 vol. %)/water mixture was prepared. Five data points with the time step of 90 s were acquired. The cuvette was irradiated with blue LED for 1 min and five data points with the time step of 90 s were acquired. Standard deviation of zeta-potential for each sample was calculated based on three measurements.

3.12. Photocatalytic oxidation of PEDOT:PSS by K-PHI in water

A suspension of K-PHI (12.5 mg) in aqueous solution of PEDOT:PSS (2 mL, 1.3 wt. %) was degassed by freeze-pump-thaw method (3 times) and the headspace of the reactor was refilled with argon. Reaction mixture was stirred under blue light irradiation for 24 h. K-PHI was separated by centrifugation, washed with deionized water (3 x 2 mL) and dried in vacuum (65 °C, 7 mbar). Two control measurements were conducted:

1. To investigate the influence of O_2, the reaction mixture with the same composition was prepared without subsequent degassing and stirred under blue light irradiation for 24 h. Workup was performed similar to the experiment under Ar.
2. To investigate the influence of light, reaction mixture with the same composition was prepared and stirred in the dark for 24 h. Workup was performed similar to the experiment under Ar.

4. Synthesis of carbon nitrides

K-PHI

K-PHI was prepared according to the reported procedure.[2] A blend of potassium chloride (2.75 g), lithium chloride (2.25 g), and 5-aminotetrazole mohonydrate (1.21 g) was grinded in a ball mill at a frequency 25 Hz for 5 min. The flour-like powder was transferred to the porcelain crucible and heated under nitrogen flow (5 L min^{-1}) using the following program: 1) heating from room temperature to 550 °C within 4 h, 2) calcination at 550 °C for 4 h. The crucibles were spontaneously cooled to room temperature. The cake and deionized water (100 mL) were brought together in a beaker and stirred at room temperature for 3 h. Solid was separated by centrifugation (4000 min^{-1}, 15 min) followed by washing with water (3x2 mL) and drying in vacuum (20 mbar) at 55 °C for 15 h.

Material characterization was reported.[3,2,4]

mpg-CN

Cyanamide (3.0 g) and Ludox® HS-40 (7.5 g) were mixed in a 10 mL glass vial. The mixture was stirred at room temperature for 30 min until cyanamide has completely dissolved. The resultant solution was stirred at +60°C for 16 h until water has completely evaporated. Magnetic stir bar was removed and white solid was transferred to the porcelain crucible and heated under N_2 flow (5 L min^{-1}) in the oven. The temperature was increased from room temperature to 550°C within 4 h and maintained at 550°C for 4 h. The crucible was spontaneously cooled to room temperature. The solid from the crucible was briefly grinded in the mortar and transferred to the polypropylene bottle. A solution of $(NH_4)HF_2$ (0.24 g mL^{-1}, 50 mL) was added and suspension was stirred at room temperature for 24 h. The solid was filtered, thoroughly washed with water, once with ethanol and dried in vacuum (55°C, 20 mbar) overnight.

Material characterization was reported.[5]

g-CN

Dicyandiamide (15 g) was calcined in a porcelain crucible under flow of N_2 (5 L min^{-1}) using the following settings: 1) heating from room temperature to 600 °C within 4 h, 2) at 600 °C for 4 h. The solid was grinded in a mortar. Yield: 5.18 g.

Na-PHI

A mixture of melamine (1.5 g) and sodium chloride (15 g) was grinded in a ball mill at a frequency 25 Hz for 2 min. The flour-like powder was transferred to the porcelain crucible and heated under nitrogen flow (5 L min^{-1}) using the following program: 1) heating from room temperature to 550 °C

for 4 h, 2) calcination at 550 °C for 4 h. The crucibles were spontaneously cooled to room temperature. The powder and deionized water (100 mL) were brought together in a beaker and stirred at room temperature for 3 h. Solid was separated by centrifugation (5500 min^{-1}, 15 min) followed by washing with water (3x40 mL) and drying in vacuum (20 mbar) at 55 °C for 15 h.

Material characterization was reported.[6]

5. Fabrication of FTO electrodes, spray coating of the electrodes with the materials, study of response to different stimuli and data processing

5.1. Preparation of K-PHI:PEDOT:PSS mixture

Solution of K-PHI nanoparticles in water (5 mg mL^{-1}) was prepared by sonicating K-PHI powder (25 mg) in deionized water (5 mL) for 10 min. K-PHI:PEDOT:PSS mixture of desired composition was prepared simply mixing a calculated amount of K-PHI nanoparticles in water (5 mg mL^{-1}) and a solution of PEDOT:PSS in water (1.3 wt. %) (Figure S2). Blends of other semiconductors and PEDOT:PSS dispersion in water with desired semiconductor content (ω) were prepared by the same procedure using amount of materials listed in Table S3. Responsivity of K-PHI:PEDOT:PSS composites to external stimuli was studied using two types of devices.

5.2. Type A FTO electrode.

For high throughput tests, a notch was curved on the piece of FTO conductive glass (30 x 20 mm) (Figure S1). One drop (10 µL) of K-PHI:PEDOT:PSS water mixture was drop casted on the FTO electrode type A. Water was evaporated by heating the electrode with air gun (Figure S3). A chamber and a setup for studying responses of the FTO electrode type A with deposited drop of K-PHI:PEDOT:PSS composite are shown in Figures S4,5. Response of the K-PHI:PEDOT:PSS hybrid nanocomposite to temperature (Figure S9) was studied by heating the chamber with the air gun. Temperature versus time was recorded using LabView based software developed at the electric workshop of the institute. I-V curve of K-PHI:PEDOT:PSS composite (Figure S10) was performed by changing voltage stepwise from 0.8 V to 5.5 V, from 5.5 V to -5.5 V and from -5.5 V to -0.8 V with step 0.1 V. The ampere meter reading were taken manually at each point.

Response (δR) of the material was calculated according to the equation:

$$\delta R = \frac{R_L - R_D}{R_D} \cdot 100\%$$

where R_D – resistance of the electrode in dark, Ohm; R_L – resistance after illuminating the electrode with light for 10 s, Ohm.

One cycle of the K-PHI:PEDOT:PSS hybrid nanocomposite response in air has been chosen for data fitting. The ‘light’ cycle was fitted with one-exponential function (R^2 = 0.99996, Figure S6):

$$R_{norm} = R_0 \cdot e^{-kt} + a$$

where R_0 = –2.7705; k = 0.03095; a = 3.77244

The ‘dark’ cycle after light irradiation is ceased was fitted with one-exponential function (R^2=0.99671, Figure S6):

$$R_{norm} = R_0 \cdot e^{-kt} + a$$

where R_0 = 0.93474; k = 0.04063; a = –0.00994

or two-exponential function (R^2=0.99994, Figure S7):

$$R_{norm} = R_{01} \cdot e^{-k_1 t} + R_{02} \cdot e^{-k_2 t} + a$$

where R_{01} = 0.21208; k_1 = 2.003; R_{02} = 0.90308; k_2 = 0.02658; a = –0.12909

5.3. Type B FTO electrode.

A pattern according to Figure S11 was engraved on the FTO layer (30 x 20 mm) with a final imprint line width of 180 µm, a line separation of 1 mm and a penetration depth of 8 µm. Spray coating has been chosen as a method to create a film due to its compatibility with the substrates possessing different shape as well as having uneven surface. The latter feature is particularly important for the present study taking into account presence of notch on the surface of the electrode. Air brush equipped with ink reservoir (5 mL) was connected to compressed air (2 bar). A patterned FTO electrode was sonicated in deionized water for 20 s, washed with acetone and dried under flow of air. Before applying coating it was ensured that the electric resistance of the FTO electrode type B >100 MΩ (Figure S12). Nonconductive side of the patterned FTO electrode was placed on preheated to +35 °C plate. It has been reported that high substrate temperature (+125°C) in PEDOT:PSS film deposition by spray coating is beneficial to obtain smoother films.[7] However, we found that heating above +60°C leads to cracking of the patterned FTO glass. Therefore, in the developed method substrate temperature was limited to +35°C. A mixture of K-PHI:PEDOT:PSS in water was spray coated on the surface of the patterned FTO electrode while constantly monitoring resistance. Spray-coating parameters were the following: nozzle tip to substrate distance 5 cm; air pressure 2 bar; spray time 5 min; drying time 60 s. The procedure of spray coating was performed continuously until the desired resistance of the film was achieved. The electrode was disconnected from the ohmmeter and stored in glass vial on air. To extended the lifetime of the electrodes we limited current to <7 µA by fabricating the photoredox device with initial resistance 160 kOhm. The chamber for electrode type B tests was assembled sandwiching PTFE frame and 2 silicon gaskets between two soda-lime glass windows (Figure S20). The chamber was equipped with two electrodes to connect the device to a supply of constant voltage and an Amperemeter, as well as reference O_2 sensor and temperature sensor (Figure S21). To investigate the response of K-PHI:PEDOT:PSS hybrid nanomaterial to oxygen, a complex program was developed (Figure S22). We altered cycles of purging the chamber with pure N_2 and N_2/O_2 mixture. O_2 concentration was changed from 1 vol. % to 100 vol. %. Second, to trigger

formation of the long-lived radical of K-PHI, the chamber was irradiated with light for 10 s followed by a dark cycle lasting 60 s. In total, 540 cycles were performed. The chamber was sequentially flushed with N_2/O_2 mixture with calculated O_2 concentration followed by a cycle of flushing the chamber with pure N_2. Duration of each period is 2100 s, equivalent to 30 cycles of LED ON/OFF (30 x (10 s + 60 s)). Response of K-PHI:PEDOT:PSS to different environment and light irradiation was studied applying constant voltage (1 V that is equivalent to 83 mV cm^{-1} taking into account the length of the notch). Gas mixture flow was set to 9.00 ccm min^{-1}.

In the experiments using type B electrode, current (I) was recorded. Resistance (R) of the electrode was calculated using Ohm's law:

$$R = \frac{U}{I} = \frac{1}{I}$$

where *U* – applied potential, V; *I* – current, A.

LabTalk scripts were used to process raw data, perform fitting and calculate respective lifetimes (τ_1, τ_2, τ_3).

Calculation of τ_1:

1. A data set composed of *t* and *R* values corresponding to the data points at the end of cycles No. 30, 60, 120, 180, 240, 300, 360, 420, 480, 540 was generated.
2. The data set was fitted with the exponential function
$$y = y_0 + A_1 \cdot e^{-\frac{x}{t_1}}$$
3. Half-life τ_1 was calculated using the equation
$$\tau_1 = \ln(2) \cdot t_1$$

Calculation of τ_2:

1. Eighteen data sets composed of t and R values corresponding to the data points at the end of cycle with the constant O_2 concentration.
2. Each data set was fitted with the exponential function
$$y = y_0 + A_1 \cdot e^{-\frac{x}{t_1}}$$
3. Half-life τ_2 was calculated using equation
$$\tau_2 = \ln(2) \cdot t_1$$

Calculation of τ_3:

1. 1080 data sets (540 data sets for Light ON and 540 data sets for Light OFF part of the cycle) composed of t and R values were generated
2. Each data set was fitted with the exponential function

$$y = y_0 + A_1 \cdot e^{-\frac{x}{t_1}}$$

3. Half-life τ_3 was calculated using equation

$$\tau_3 = \ln(2) \cdot t_1$$

4. Average < τ_3> and standard deviation were calculated for the cycles with constant O_2 concentration.

6. Supplementary discussion

6.1. Steady-state fluorescence

Photoluminescence (PL) spectra (Figure S39) are composed of peaks of individual materials. Nevertheless, PL intensity of K-PHI:PEDOT:PSS composite is two times lower compared to K-PHI, suggesting that radiative recombination of excitons is suppressed due to formation of K-PHI long-lived radical.

6.2. EPR study at variable temperature

K-PHI

EPR spectra of K-PHI recorded in the range of temperature 300-90K exhibit one line with g-factor 2.003 that can be fitted with one Lorentzian derivative (Figure S24). Signal amplitude increases by ca. 3 times, while specific concentration of polarons increases ca. 3 times, from $(2.2\pm0.2)\cdot10^{16}$ to $(6.8\pm0.3)\cdot10^{16}$ g^{-1}, upon cooling. In this view, behavior of K-PHI is similar to conductive polymers rather than inorganic semiconductors.[8,9] Both parameters indicate paramagnetic ground state of K-PHI. At the same time, linewidth, 5.8±0.3 G, does not depend on temperature (Figure S29), therefore dipolar, unresolved hyperfine and exchange interactions are the main pathways for energy dissipation, while spin-lattice relaxation has negligible effect in K-PHI.[10,11]

PEDOT:PSS

EPR spectra of PEDOT:PSS acquired in the range of temperature from 300K to 90K are more complex, but can be fitted with two Lorentzian derivatives, broad and narrow, both with the *g*-factors of 2.003 (Figure S25). Such observation is in agreement with earlier report for PEDOT:PSS, in which narrow line has been assigned to polarons, confined in isolated spin packets, while wide – to delocalized polarons, associated with the electrons of conductivity.[12] Upon cooling the amplitude of the EPR signal increases >40 times (Figure S27). Our results are in agreement with the reported earlier for PEDOT:PSS and suggest that upon cooling polarons in PEDOT:PSS become more localized.[10] Furthermore, analysis of the integrated peak area shows that total specific concentration of polarons in PEDOT:PSS increases ca. 2 times, from $(5.1\pm0.97)\cdot10^{18}$ to $(10\pm2)\cdot10^{18}$ g^{-1}, upon cooling (Figure S28). Separate integration of the components revealed that narrow component is solely responsible for increased concentration of polarons in the sample upon cooling, presumably, at the expense of bipolarons. Linewidth of the narrow component remains constant, i.e. 2.96±0.26 G, in the range of temperature 300K-90K, while the linewidth of the broad component decreases ca. 2 times, from 19.7±1.4 to 7.65±0.53 G (Figure S29). These results indicate that followed by excitation with the electromagnetic radiation, electrons in PEDOT:PSS dissipate energy via two pathways – spin-lattice relaxation and spin-spin

interaction.[10,11] At lower temperature, polarons become more localized, the mobility decreases, as evidenced by narrower linewidth at cryogenic temperature.

K-PHI:PEDOT:PSS

EPR spectra of K-PHI:PEDOT:PSS composite can be also fitted with two Lorentzian derivatives with *g*-factors of 2.003 (Figure S26). Similar to PEDOT:PSS, amplitude of the EPR signal increases ca. 13 times upon cooling (Figure S27). In K-PHI:PEDOT:PSS, total specific concentration of polarons increases ca. 8 times, from $(3.8\pm0.6)\cdot10^{17}$ to $(29\pm2)\cdot10^{17}$ (Figure S28). Although total specific concentration of polarons in K-PHI:PEDOT:PSS is lower compared to pure PEDOT:PSS, temperature dependence is much more pronounced compared to both K-PHI and PEDOT:PSS. Unlike to pure PEDOT:PSS, specific concentration of both, highly mobile polarons and localized polarons, strongly depends on temperature (Figure S28). Therefore, K-PHI nanoparticles facilitate interconversion between the localized and highly mobile polarons upon cooling. Linewidth of the narrow component remains nearly constant, i.e. 2.88±0.24 G, in the range of temperature 90-300K that is similar to pure PEDOT:PSS (Figure S29). However, linewidth of the broad component decreases by 40%, from 23.1±4.0 to 14.6±1.1 G, in the range of temperature 90-300K.

6.3. EPR study under light irradiation.

K-PHI

Upon irradiation with light, K-PHI powder did not show significant change of the signal amplitude, neither linewidth (Figure S30, S32).

PEDOT:PSS

PEDOT:PSS demonstrated clear response to irradiation with blue light as evidenced by increasing of the signal amplitude 2 times immediately upon irradiation with light followed by slow recovery to the initial magnitude in dark under ambient conditions (Figure S31). Deconvolution of the signal into broad and narrow components revealed that light irradiation equilibrates, at some extent, mobility of polarons toward the mean value – the linewidth of the broad component narrows by ca. 17%, from 19.8 G to 16.4 G, while at the same time the linewidth of the narrow component broadens by ca. 6%, from 3.3 G to 3.5 G (Figure S33). Concentration of highly mobile polarons (broad component of the EPR spectrum) increases by ca. 5%, from $(4.2\pm0.3)\cdot10^{18}$ to $(4.4\pm0.4)\cdot10^{18}$ g^{-1}, while concentration of localized polarons (narrow component of the EPR spectrum) increases by ca. 8%, from $(1.2\pm0.05)\cdot10^{17}$ to $(1.3\pm0.06)\cdot10^{17}$ g^{-1} (Figure 33). Increasing concentration of polarons we explain by light-induced electron transfer between PEDOT neutral state and dication (bipolaron) that will be also proved by transient absorption spectroscopy (see below). Linewidths of broad and narrow components as well as the concentration of polarons

associated with these components return to the original values within ca. 93 h (Figure S33), suggesting that response of PEDOT:PSS to light is reversible.

K-PHI:PEDOT:PSS

K-PHI:PEDOT:PSS shows pronounced response to light irradiation with the signal amplitude increasing immediately after sample irradiation with blue light followed by gradual recovery in dark (Figure 4b). However, linewidth of the broad component decreases ca. 38%, from 29±1 G to 18±0.5 G, while the linewidth of the narrow component increases ca. 32%, from 3.4±0.05 G to 4.5±0.4 G (Figure S34), suggesting that K-PHI effectively facilitates localization of polarons upon light irradiation. Concentration of highly mobile polarons (broad component) in K-PHI:PEDOT:PSS increases ca. 4 times, from $(4±1)·10^{17}$ to $(16±6)·10^{17}$ g^{-1}, while concentration of localized polarons (narrow component) increases ca. 4 times, from $(4±0.7)·10^{16}$ to $(19±7)·10^{16}$ g^{-1}, suggesting that addition of K-PHI nanoparticles to PEDOT:PSS enhances selectivity of localized polarons formation via electron transfer between PEDOT neutral states and bipolarons (Figure S34). In this view, K-PHI nanoparticles serves as a catalyst selectively enabling one reaction pathway. Although, upon light irradiation total specific concentration of polarons increases ca. 3 times, from $(4.5±1.3)·10^{17}$ to $(18±7)·10^{17}$ g^{-1}, the overall electric conductivity of the material decreases, as described above (Figure 2d). Given that conductivity of the material is proportional to the concentration of charge carriers as well as their mobilities, we conclude that decreases of polarons mobility is responsible for decrease of PEDOT:PSS conductivity triggered by light irradiation.

6.4. AFM study

Diameter of K-PHI nanoparticles range from 30 to 400 nm that is consistent with hydrodynamic diameter determined by DLS.[13] Morphology of the films has been characterized by atomic force microscopy (AFM) (Figure S17-19). Roughness of K-PHI:PEDOT:PSS film was determined to be 44±8 nm. Average±std were calculated based on the data obtained from three regions with field of view 10 x 10 μm. An example of K-PHI:PEDOT:PSS film AFM image is shown in Figure S19. The morphology of the film is represented by K-PHI nanoparticles (diameter 30-400 nm) incorporated in the agglomerated array of PEDOT:PSS nanoparticles (diameter 20-50 nm). Analyses of 18 articles on preparation of PEDOT:PSS films by different techniques clearly point that roughness of K-PHI:PEDOT:PSS films studied in this work is higher (Table S5). Higher roughness of K-PHI:PEDOT:PSS films are explained by the presence of 100-300 nm K-PHI particles with the content of ca. 30 wt. %, which impedes fabrication of smoother films. Nevertheless, higher roughness of K-PHI:PEDOT:PSS films might be beneficial for enhancing the sensitivity of the composite – it increases the surface area, which, in turn, facilitates the effective quenching of $(K\text{-}PHI)^{•-}$ by O_2.

6.5. TAS

K-PHI

It should be noted that the lifetime of K-PHI radical greatly exceeds the duration of the experiment (300 µs). Therefore, the differential absorption is determined versus the absorption of the 'green' K-PHI radical. Indeed, directly after photoexcitation at 387 nm, negative differential absorptions from the ground state bleach (GSB) of K-PHI around 440 nm evolve (Figure S37). Directly after photoexcitation, the differential absorption spectrum shows two bands 1) from 500 to 800 nm and 2) broad band at λ>900 nm. The first band also possess a virtual minimum at 660 nm that matches well to the absorption band at 660 nm in the steady state-absorption spectrum of K-PHI long-lived radical. Therefore, excited state absorption (ESA) in the range 440-1350 nm in fact contains contribution from the K-PHI long-lived radical. The fact that differential absorption in visible and nIR region is positive suggests that the excited state has higher extinction compared to K-PHI long-lived radical.

Within approximately 2.5 ns, the 595 nm maximum shifts to 615 nm and the formation of a maximum at around 440 nm is noted. The latter ESA does not decay back to zero within the time range of our experimental setup (lifetime >300 µs). While the remaining ESA in the visible region decays within 4 µs, the feature in the nIR transforms into a broad negative transient ranging from approximately 800 to 1300 nm after 150 ns. This feature then decays to zero within 25 µs. In fact, comparing the nIR feature to diffuse reflectance spectra of K-PHI long-lived radical we were able to assign the negative transient signal to the GSB of K-PHI centered moieties.

PEDOT:PSS

Turning to PEDOT:PSS deposited on glass slides, directly after photoexcitation at 387 nm, transients form with features in the visible region of light maximizing at 600 nm and broad ESA in the nIR region with a maximum at 1055 nm (Figure S38). Additionally, a negative signal is noted above λ<1210 nm. Both positive features decay with a lifetime close or below our temporal resolution limit. With their decay, negative differential transient absorption features form, ranging from 460 to approximately 800 nm with a minimum at 700 nm together with ESA below λ<460 nm and a shift of the 1055 nm maximum to around 1000 nm. All features then decay to zero within approximately 50 µs. The ultrafast component is assigned to intraband relaxation in higher lying bands of electrons upon photoexcitation.

Previous studies on PEDOT:PSS have shown that the neutral, polaron, and bipolaron states of PEDOT show characteristic UV/vis/nIR absorptions maximizing at around 600, 900, and 1200 nm, respectively.[14] Thus, we postulate that the negative differential absorptions observed in the visible and nIR region of the electromagnetic spectrum belong to the GSB of both neutral and bipolaron state, while the positive transient absorption at 1000 nm is assigned to the ESA of the polaron state of PEDOT. In other words, upon photoexcitation, one electron from a neutral unit within a

PEDOT chain is transferred to a dicationic (bipolaron) unit, effectively forming two mono-cationic PEDOT species.

K-PHI:PEDOT:PSS

It has to be noted that directly after exposing the pale green K-PHI:PEDOT:PSS hybrid material to the pump laser, a dark green spot forms. Within the course of around 5 minutes, this spot vanishes and transforms back to the initial pale green color. This can be repeated several times without any signs of degradation. These observations suggest that lifetime of K-PHI long-lived radicals is enhanced when the material is encapsulated into a polymer matrix.

6.6. Photocatalytic oxidation of PEDOT:PSS by K-PHI in aqueous medium

After stirring K-PHI with PEDOT:PSS, the solid was recovered by centrifugation, washed with water and characterized by measuring surface zeta-potential and FT-IR. K-PHI irradiated in the presence of PEDOT:PSS under Ar shows more negative zeta-potential compared to other samples (Figure S41b), suggesting that anaerobic environment facilitates coordination of PEDOT to K-PHI. However, in agreement with earlier reports complete transfer of PEDOT chains to K-PHI renders challenging.[15] Therefore, in our experiments only a fraction of PSS is replaced by K-PHI. As a result, zeta-potential of K-PHI particle becomes more negative due to transfer of PSS backbone that has even more negative zeta-potential compared to K-PHI (Table S4). In FT-IR spectra of recovered K-PHI, we observed disappearance of the peak at 995 cm^{-1} that earlier has been assigned to the symmetric vibrations of $N-C_2$ bonds of $K-NC_2$ moieties (Figure S41c).[16] In addition, peaks in the range 1200-1700 cm^{-1} assigned to N-H bending, C-N and C=N stretching in K-PHI became more pronounced compared to K-PHI suggesting equilibration of K-PHI structure. Overall, in the studied process a fraction of benzenesulfonic groups is substituted by coordination of PEDOT to negatively charged C_2N^- groups (and surface O^-). This process leads to transfer of PSS backbone to K-PHI, which shifts zeta-potential of K-PHI to more negative numbers. Schematically this process is depicted in Figure S41d.

7. Supplementary Figures

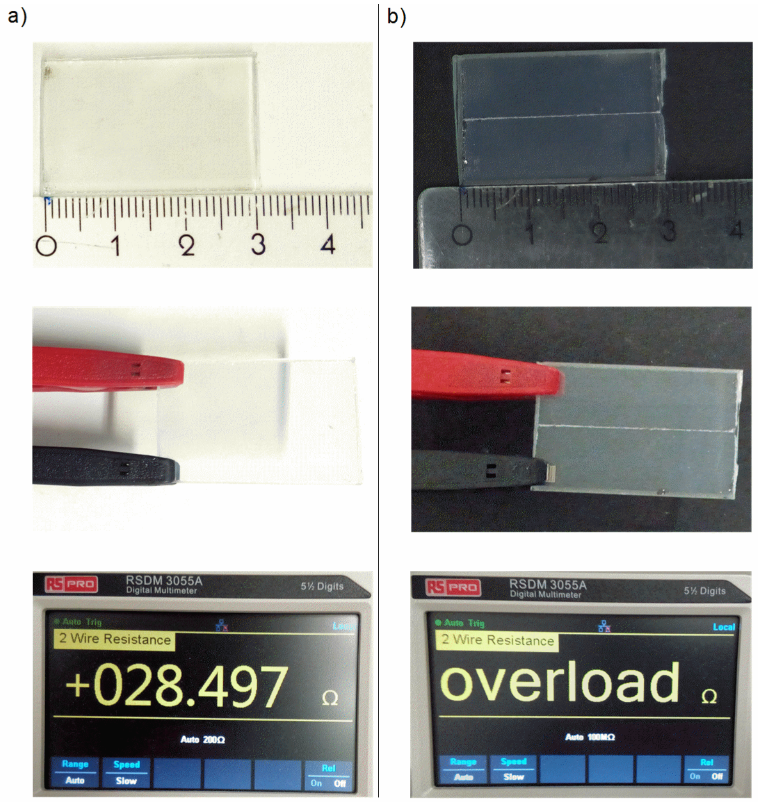

Figure S1. Preparation of FTO electrode type A. a) FTO electrode before curving the notch – appearance and electric resistance; b) FTO electrode after curving the notch – appearance and electric resistance.

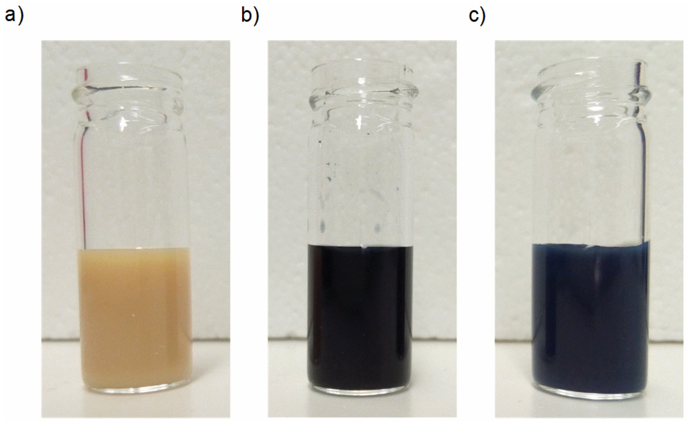

Figure S2. Appearance of K-PHI nanoparticles, PEDOT:PSS and K-PHI:PEDOT:PSS suspensions in water. a) Suspension of K-PHI in deionized water (5 mg mL^{-1}); b) PEDOT:PSS solution in water (1.3 wt. %); c) K-PHI:PEDOT:PSS mixture (1:1 by volume, 28 wt. %).

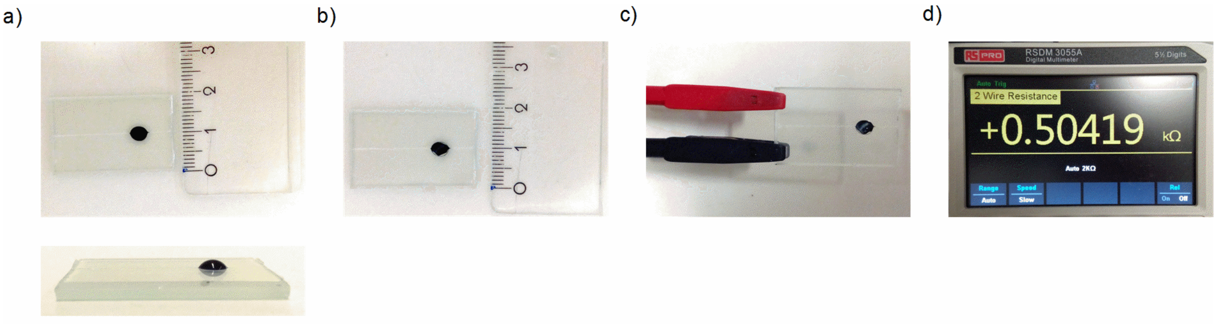

Figure S3. Fabrication of FTO electrode coated with K-PHI:PEDOT:PSS mixture. a) FTO electrode type A with a deposited drop of K-PHI:PEDOT:PSS aqueous mixture; b) FTO electrode type A after water evaporation; c) FTO electrode type A with connected crocodile clips; d) electric resistance readings.

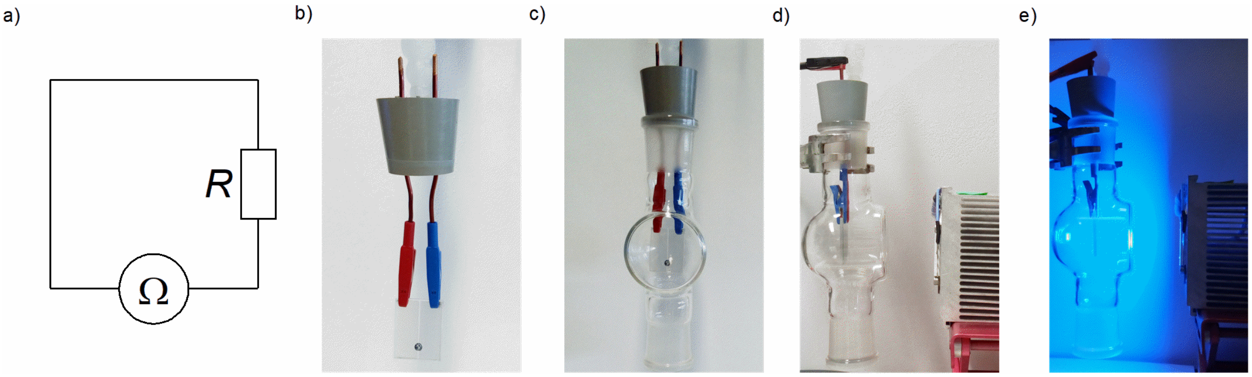

Figure S4. Electrode type A test chamber. a) Schematics of the test circuit; b) FTO electrode spray-coated with K-PHI:PEDOT:PSS mixture in the holder; c) Front view of the assembled setup under day light; d) Side view of the assembled setup under day light; e) Side view of the assembled setup under blue light irradiation.

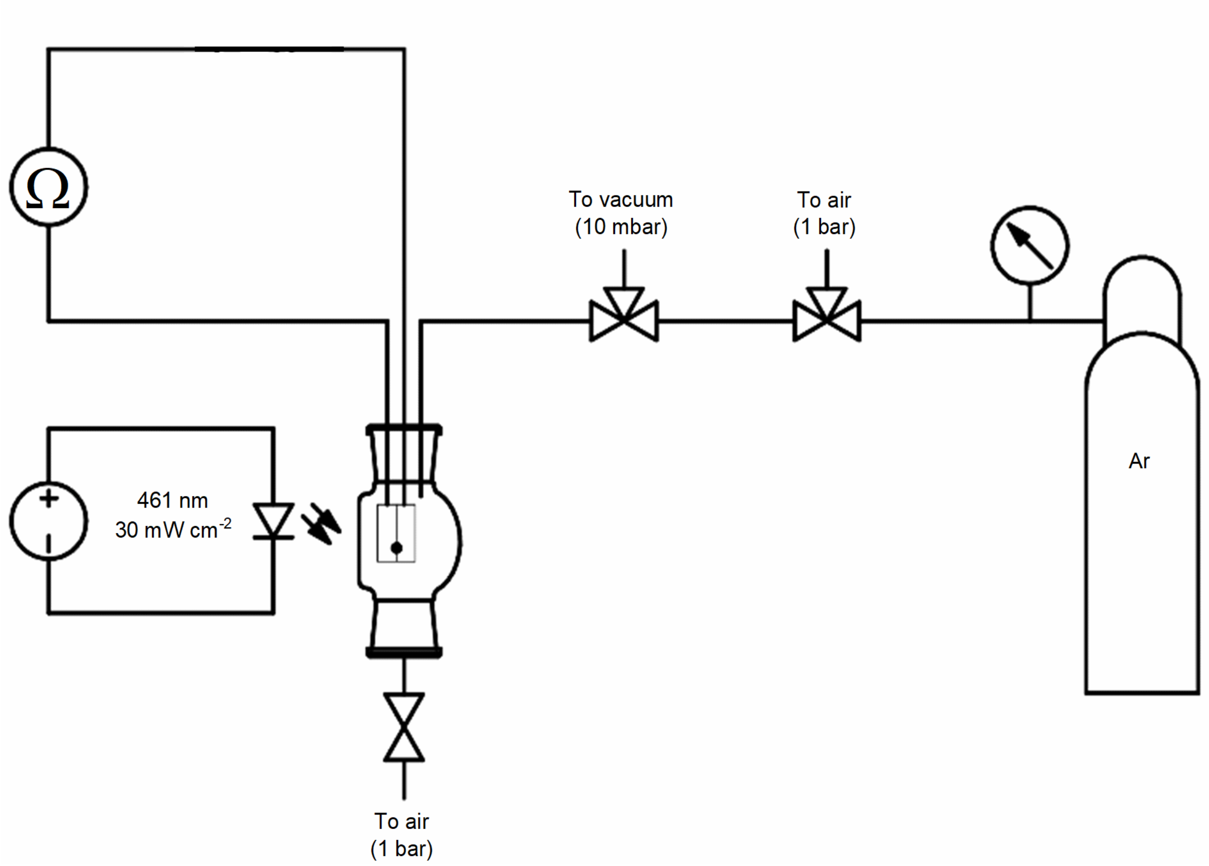

Figure S5. Schematic representation of the setup for high-throughput K-PHI:PEDOT:PSS hybrid composites tests under Ar, Air or vacuum.

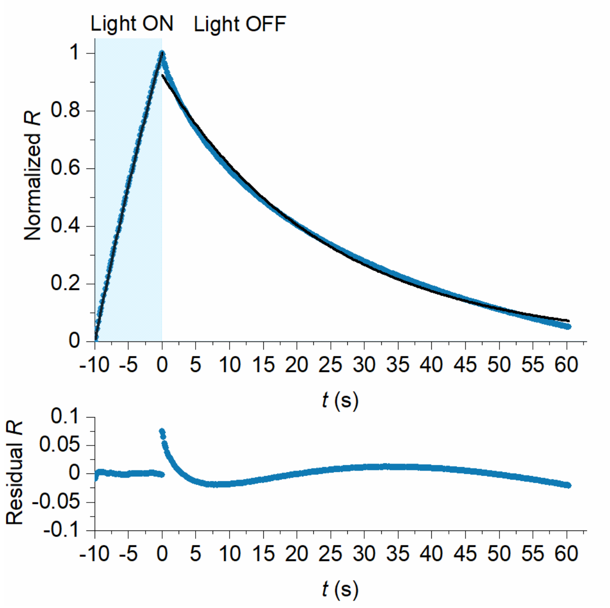

Figure S6. Fitting of K-PHI:PEDOT:PSS hybrid nanocomposite resistance change in air triggered by light irradiation. One-exponential function was used both for 'light' and 'dark' regions. Residuals are shown.

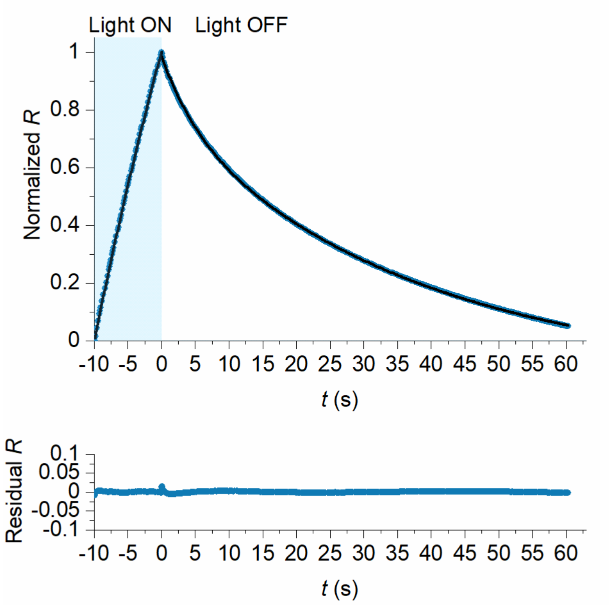

Figure S7. Fitting of K-PHI:PEDOT:PSS hybrid nanocomposite resistance change in air triggered by light irradiation. One-exponential function was used for 'light' region, two-exponential function – for 'dark' region. Residuals are shown.

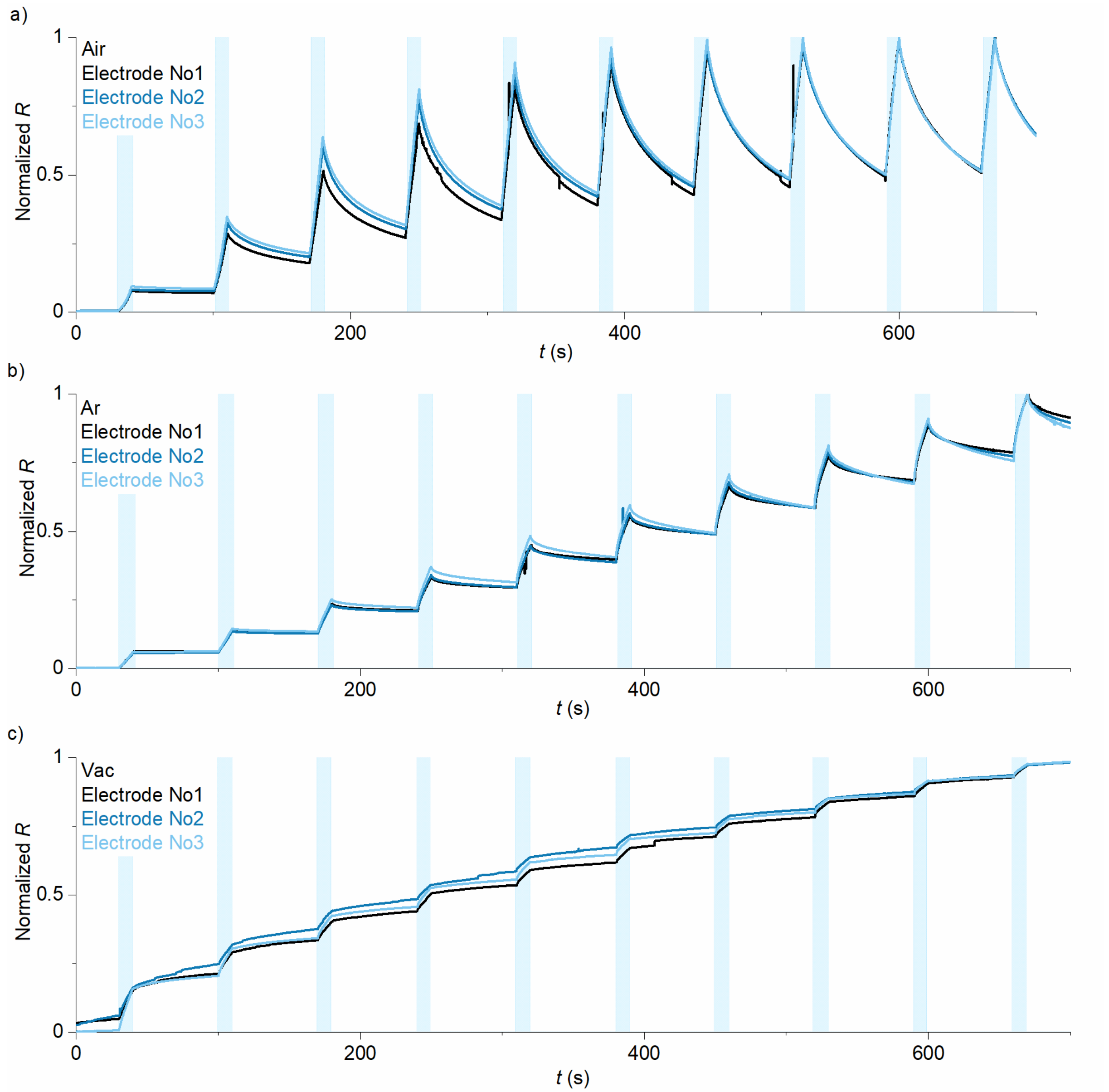

Figure S8. Reproducibility of measurements. Resistance measurements of K-PHI:PEDOT:PSS hybrid nanocomposite spray coated onto electrode type B in air (1 bar) (a), in Ar (1 bar) (b) and in vacuum (10 mbar) (c). Shaded areas denote periods of time when samples were irradiated with light. Three devices were prepared and measured sequentially under different conditions. Conditions: K-PHI content 28 wt. %, I = 30 mW cm^{-2} (461 nm), duration of the sample illumination 10 s, dark cycle 60 s.

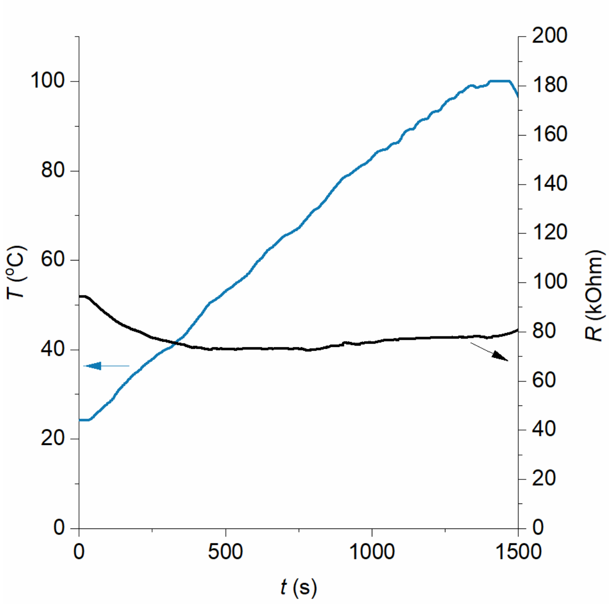

Figure S9. K-PHI:PEDOT:PSS film resistance measurements at different temperature.

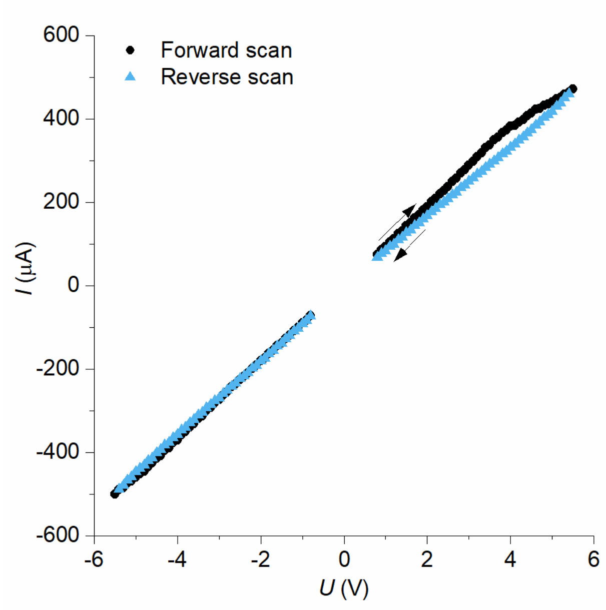

Figure S10. *I-V* curve of K-PHI:PEDOT:PSS composite film on FTO glass.

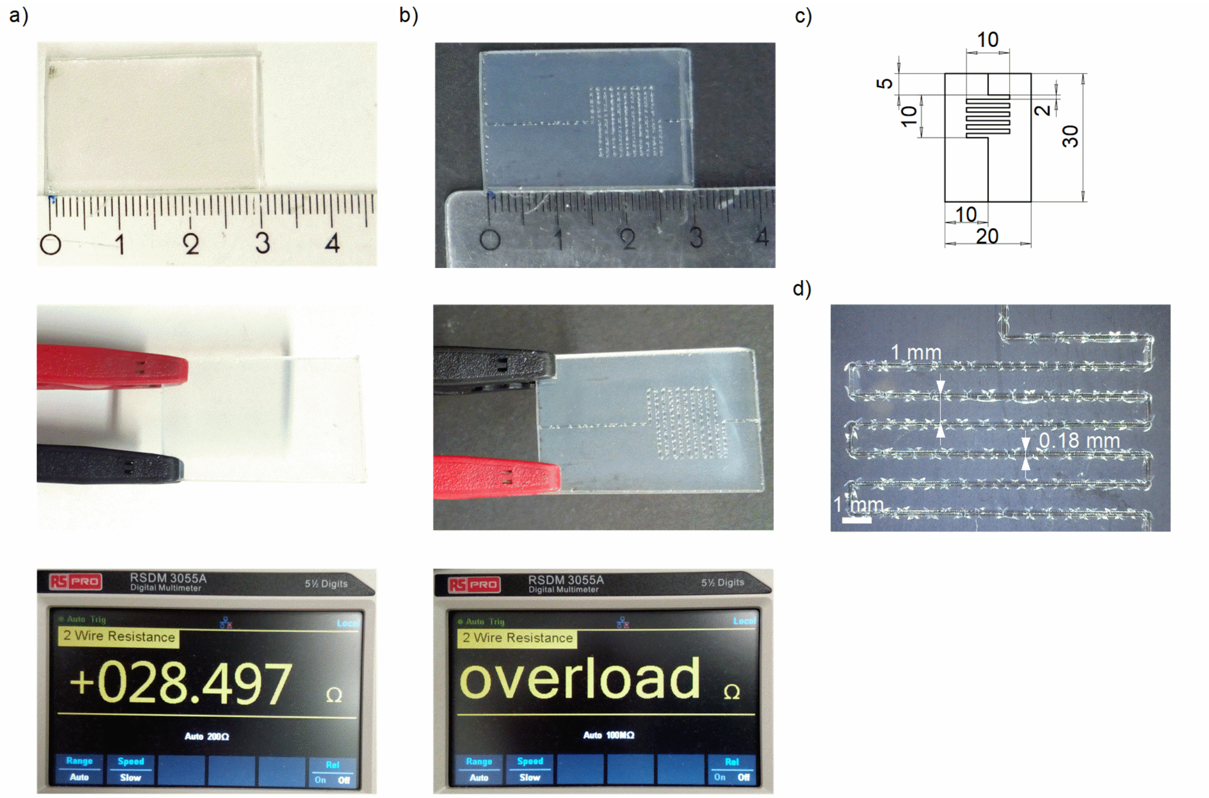

Figure S11. FTO electrode type B preparation. a) A piece of FTO glass – appearance and electric resistance; b) FTO electrode type B after curving the pattern – appearance and electric resistance; c) FTO electrode type B draft; d) Optical microscope image of the FTO electrode type B.

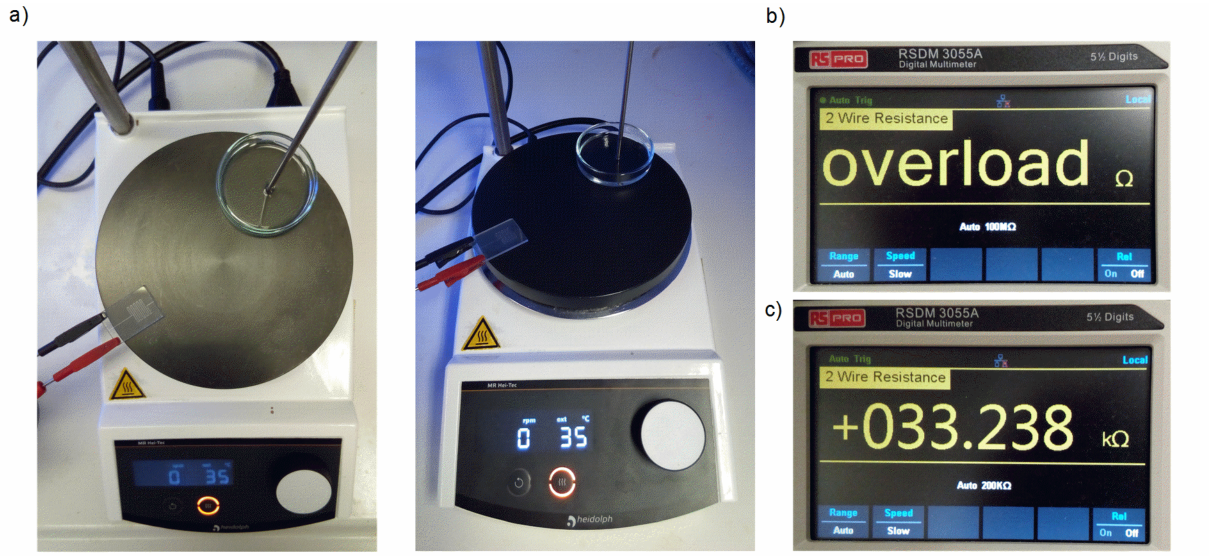

Figure S12. Spray coating of FTO electrode coated with K-PHI:PEDOT:PSS mixture. a) Setup for FTO electrode type B spray coating with K-PHI:PEDOT:PSS mixture; b) Readings of the ohmmeter of the FTO electrode type B before spray coating with K-PHI:PEDOT:PSS mixture; c) Electric resistance of the FTO electrode type B after spray coating with K-PHI:PEDOT:PSS mixture.

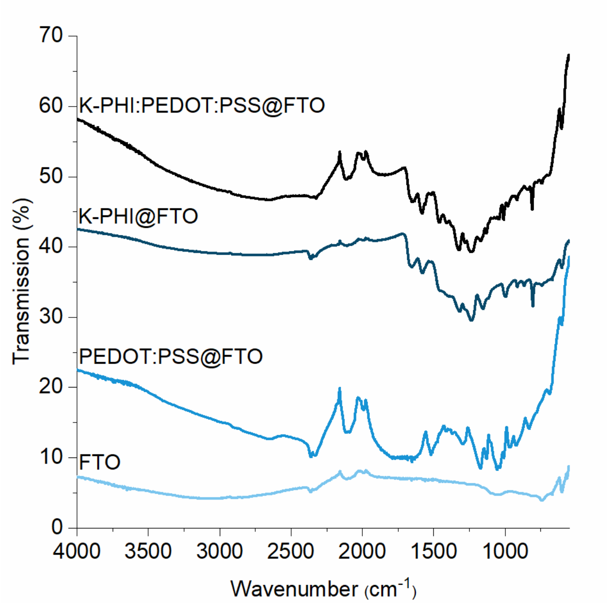

Figure S13. FT-IR spectra of materials films on FTO glass.

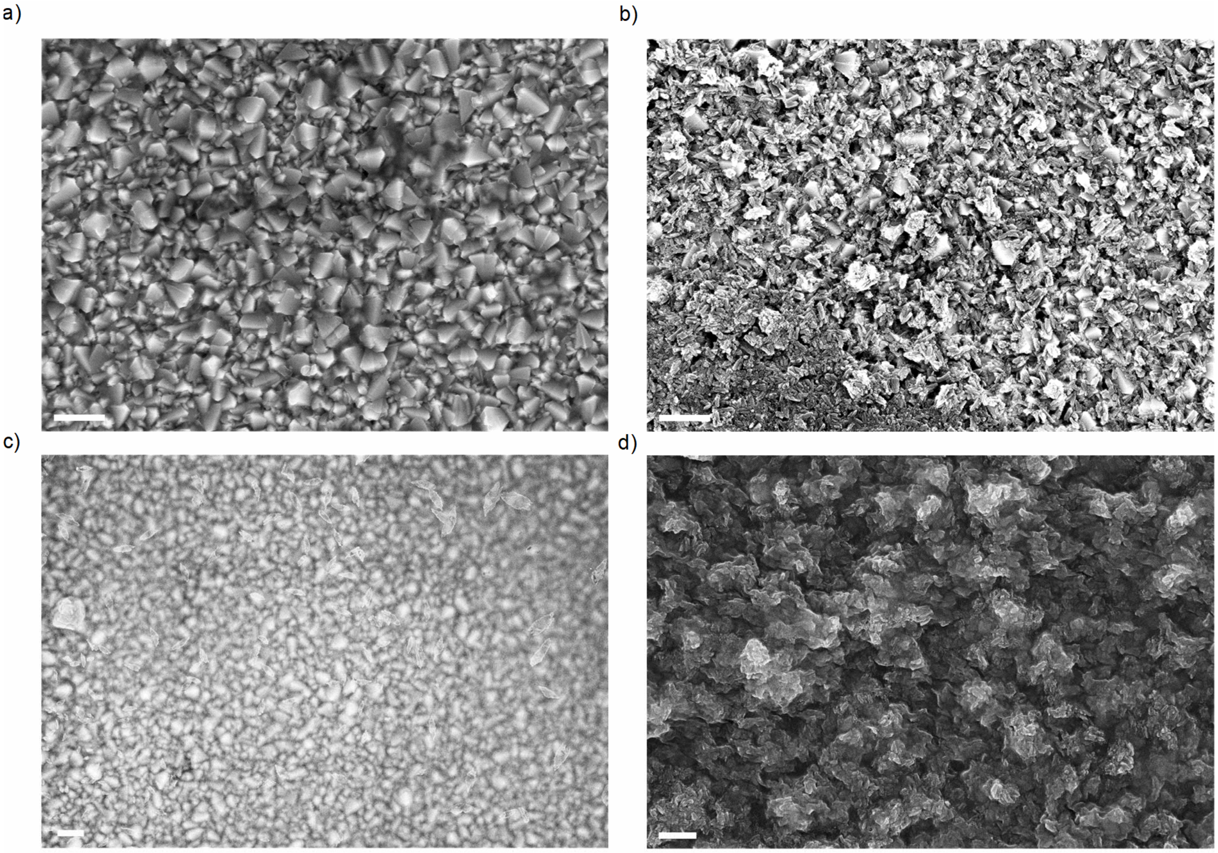

Figure S14. SEM Images of the materials films on FTO glass. Scale bar 1 µm. a) Clean FTO glass. b) K-PHI deposited on FTO glass. c) PEDOT:PSS deposited on FTO. d) K-PHI:PEDOT:PSS deposited on FTO glass.

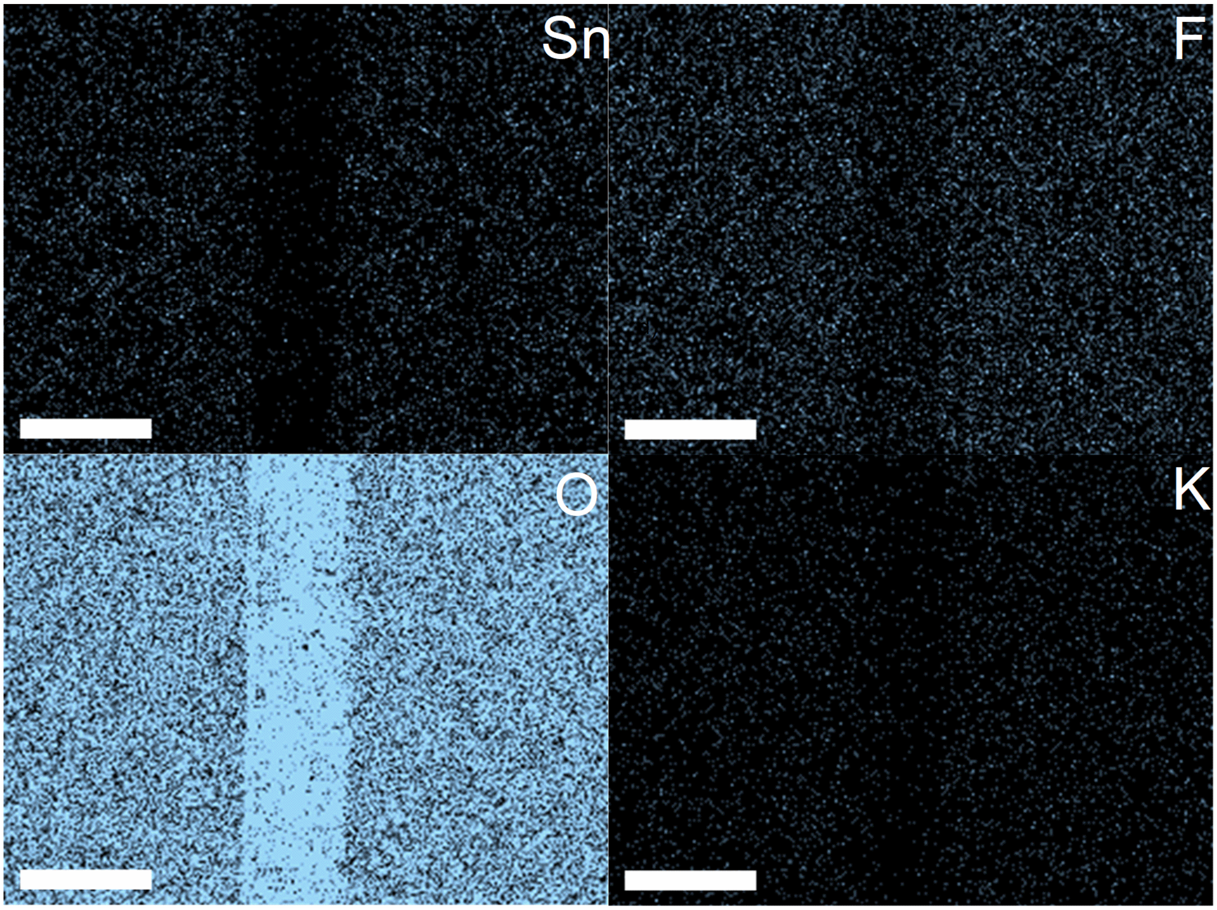

Figure S15. SEM-EDX mapping of the patterned FTO glass coated with K-PHI:PEDOT:PSS. Scale bar 250 µm.

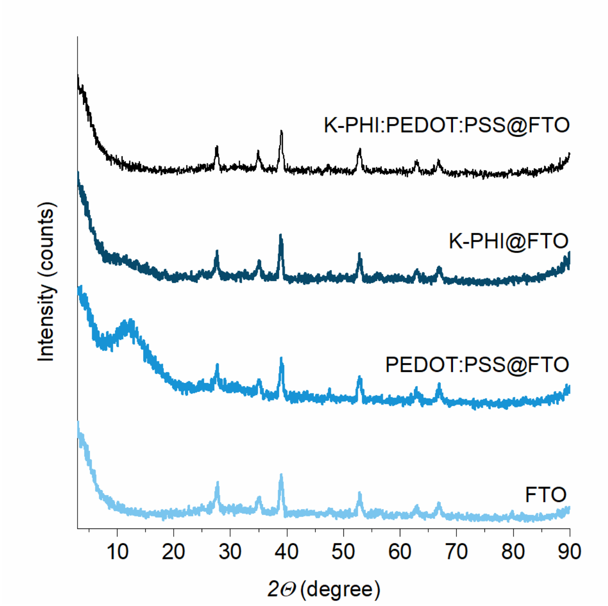

Figure S16. PXRD patterns of the materials films on FTO glass.

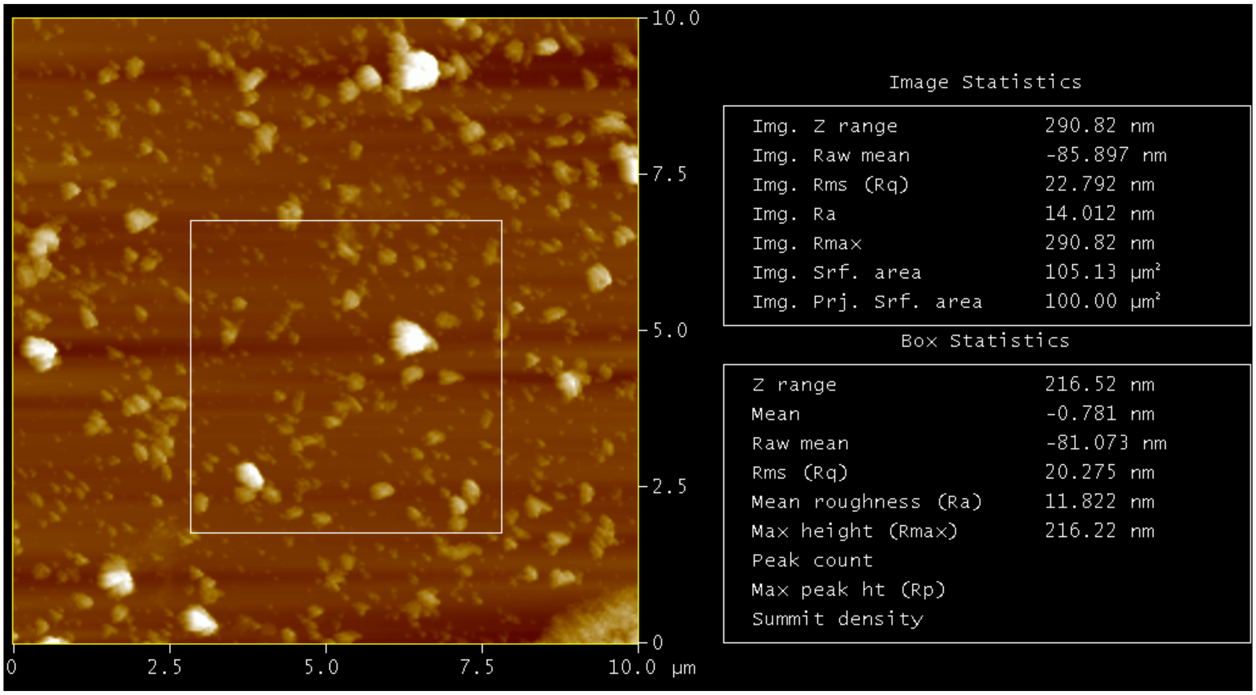

Figure S17. AFM image of K-PHI nanoparticles.

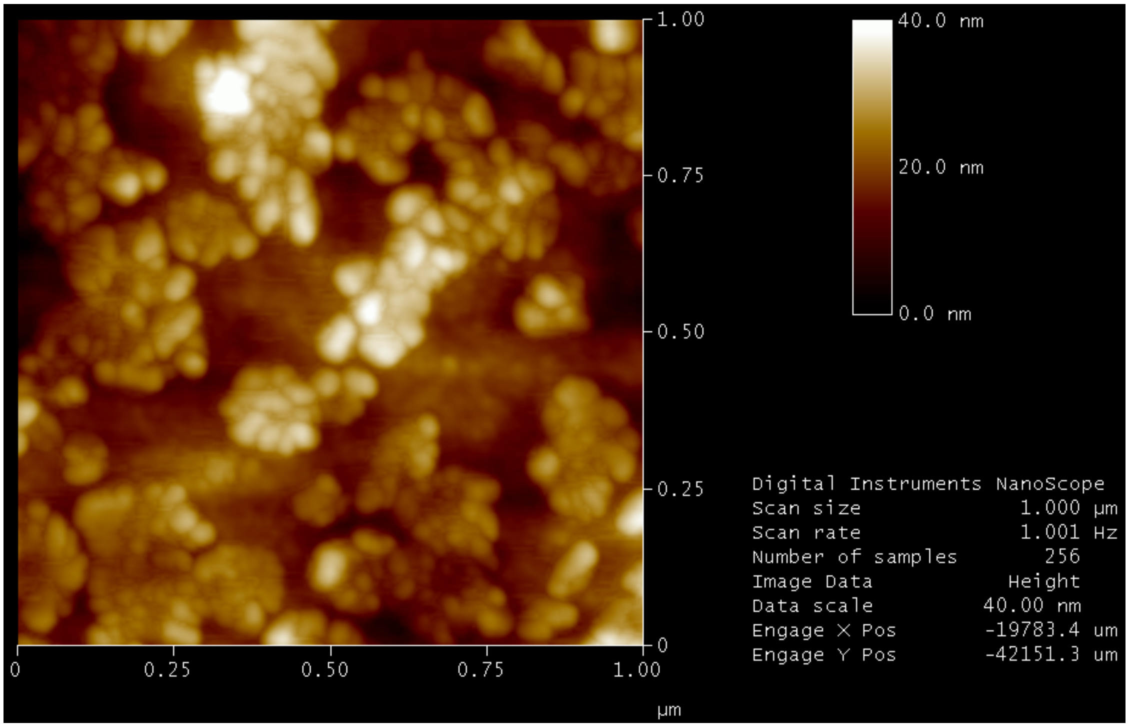

Figure S18. AFM image of PEDOT:PSS film.

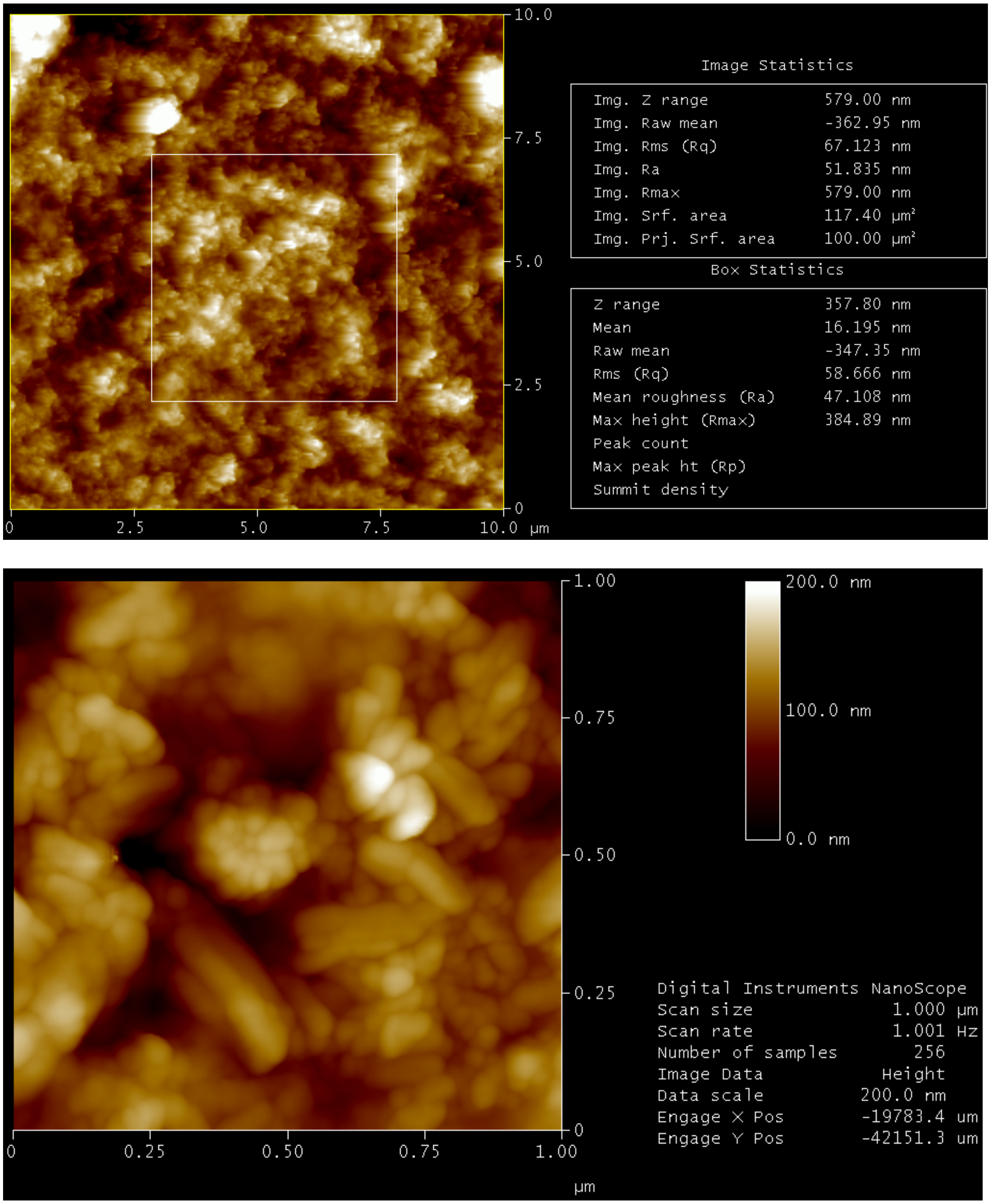

Figure S19. AFM image of K-PHI:PEDOT:PSS film.

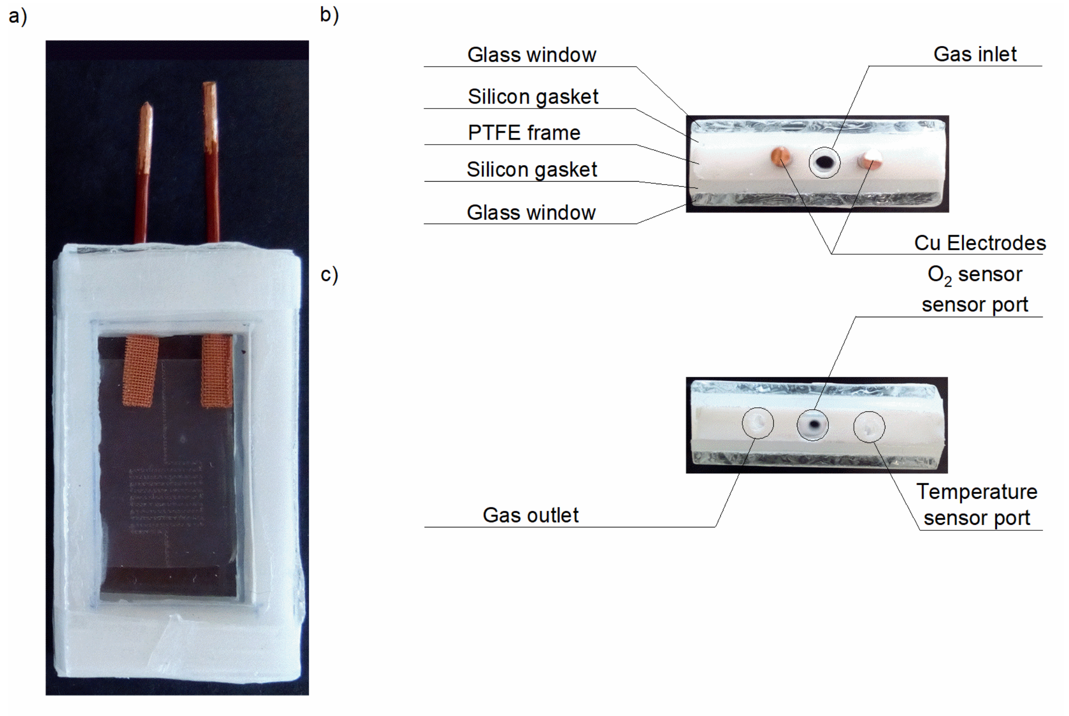

Figure S20. Chamber for testing of K-PHI:PEDOT:PSS hybrid nanocomposite films at FTO glass with simultaneous monitoring of temperature and O_2 concentration. a) Assembled chamber with FTO electrode type B; b) Top view; c) Bottom view.

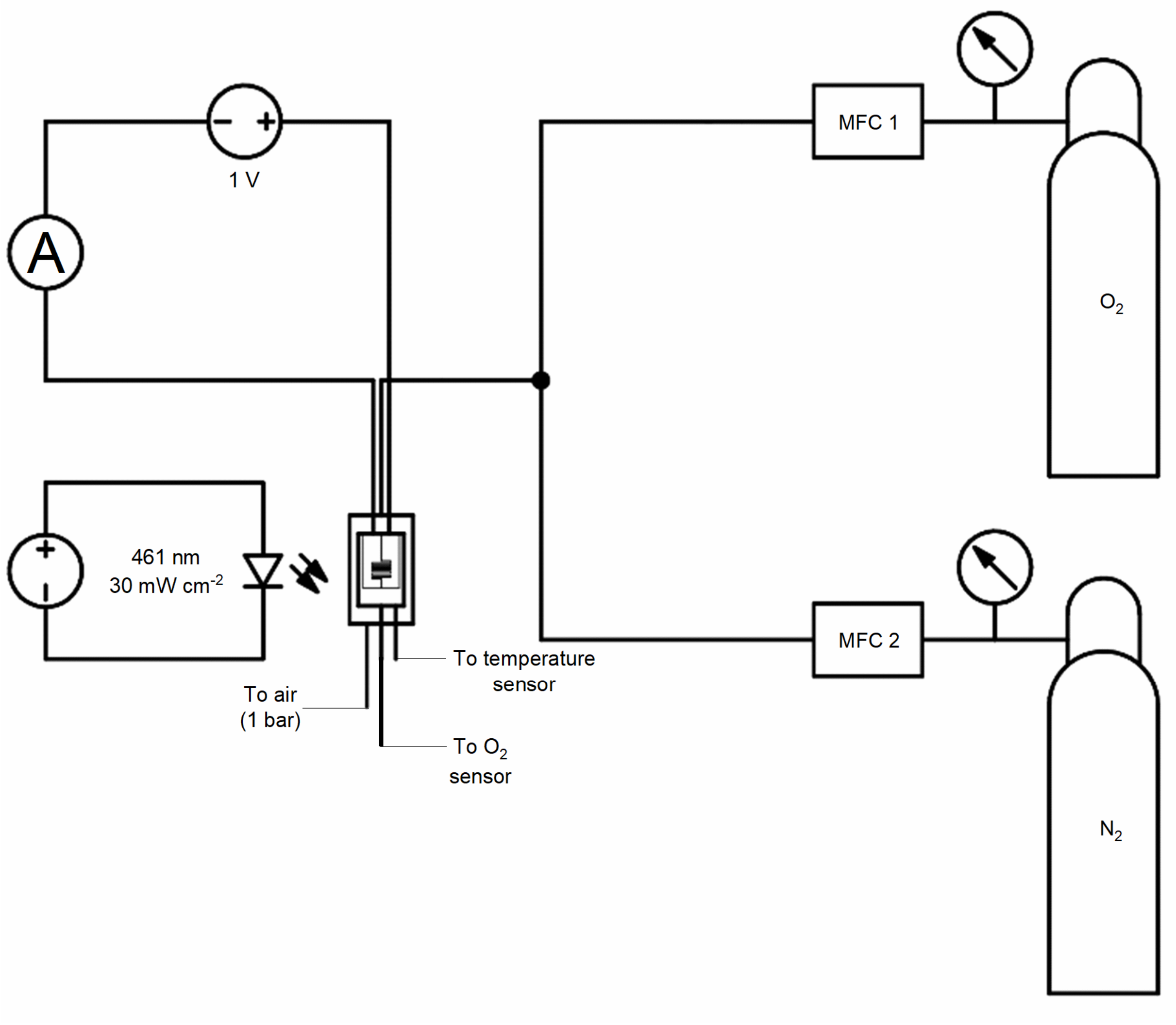

Figure S21. Schematic representation of the setup for K-PHI:PEDOT:PSS hybrid composites tests in environment with different O_2 concentration.

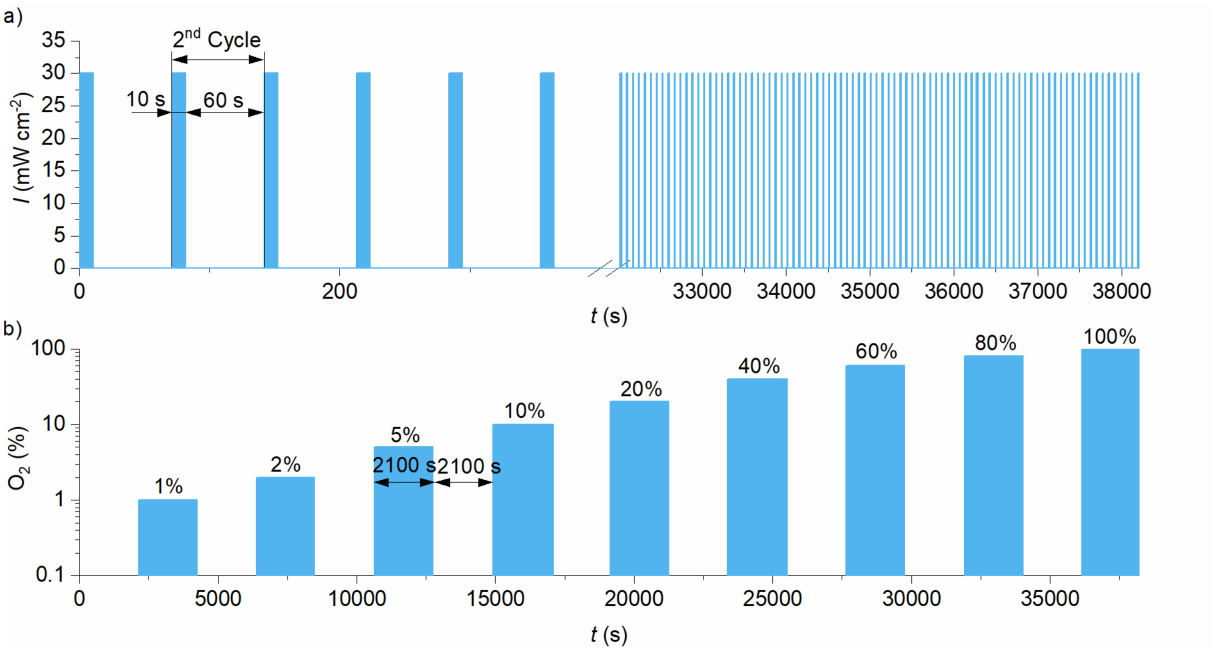

Figure S22. Schematic representation of the program developed to tests the devices. a) LED ON/OFF cycles. b) Cycles of gas composition change.

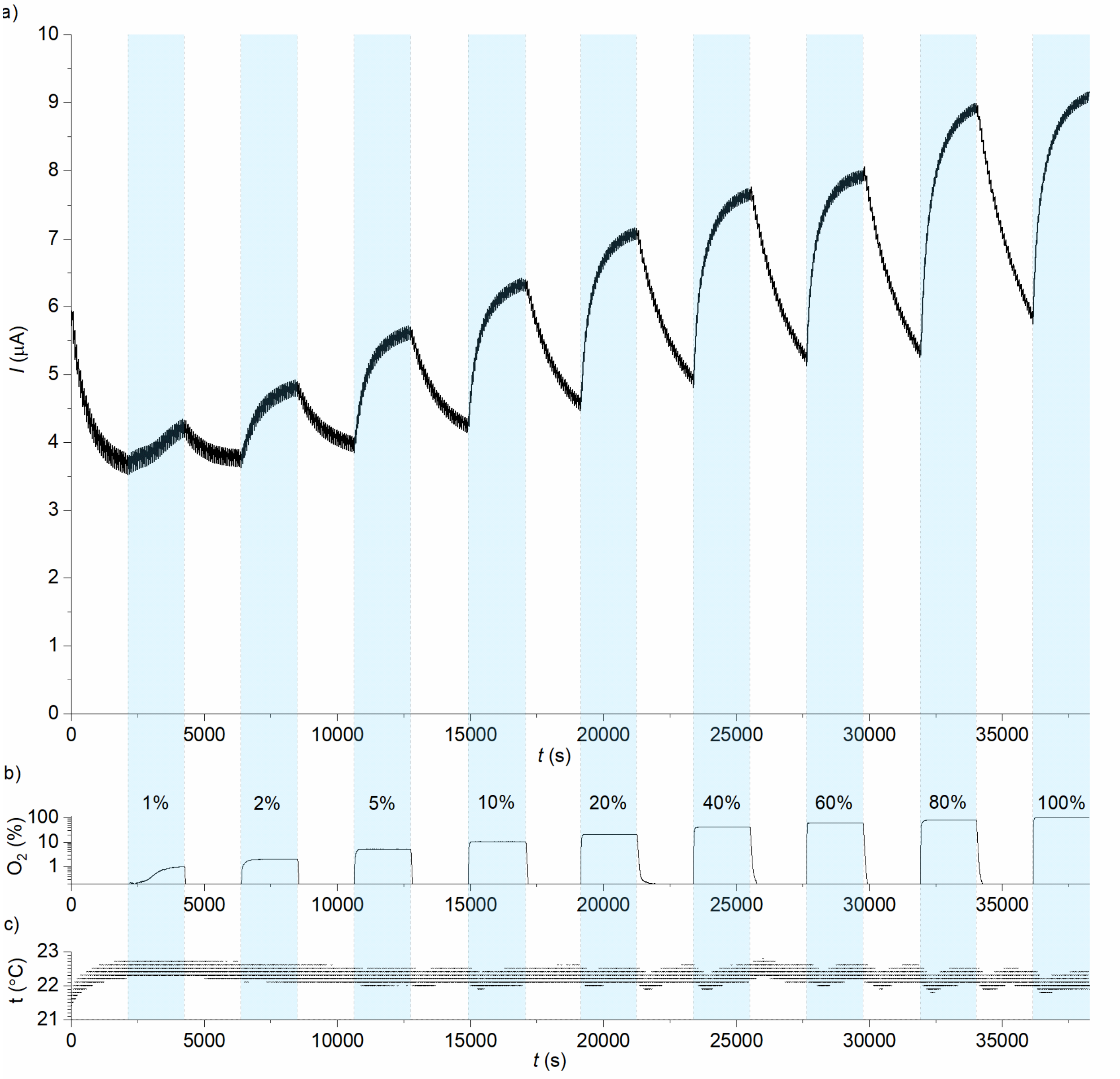

Figure S23. K-PHI:PEDOT:PSS hybrids composite tests. a) Time dependent current through the film; b) Time dependent O_2 concentration in the chamber (semi logarithmic scale); c) Gas temperature in the chamber versus time.

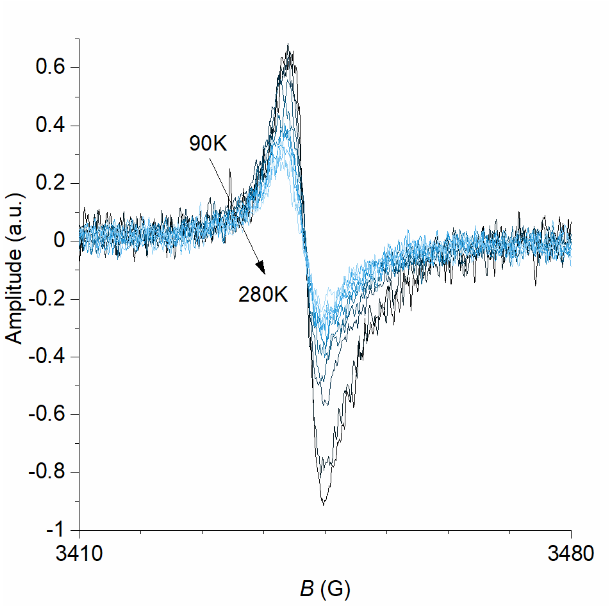

Figure S24. EPR spectra of K-PHI acquired in the range of temperature 90-280K.

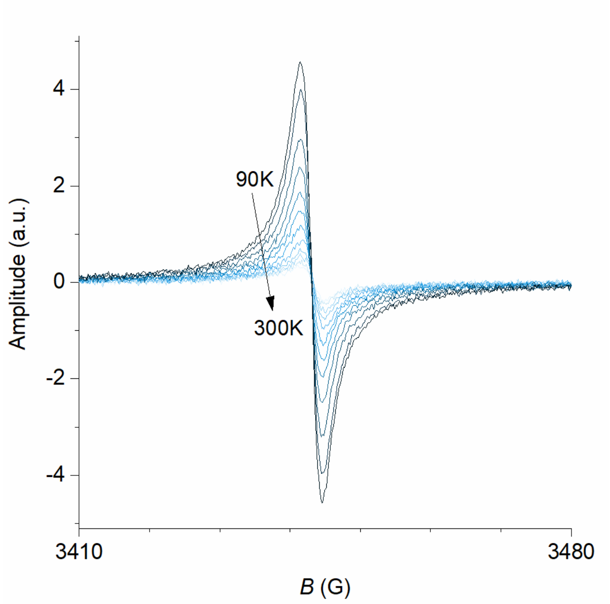

Figure S25. EPR spectra of PEDOT:PSS acquired in the range of temperature 90-300K.

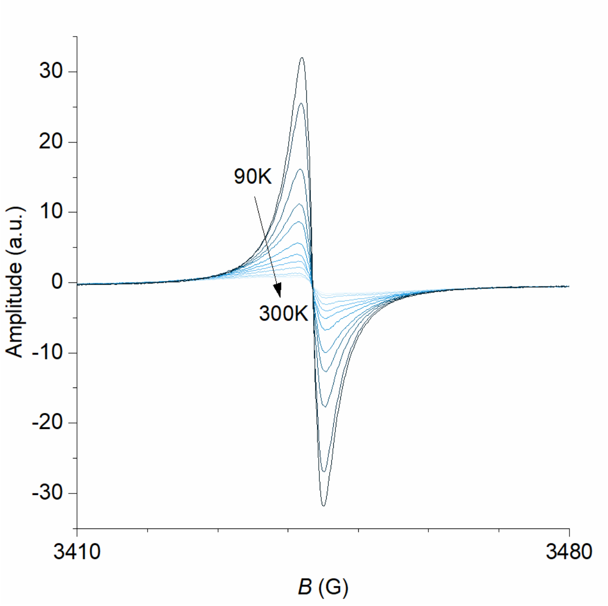

Figure S26. EPR spectra of K-PHI:PEDOT:PSS acquired in the range of temperature 90-300K.

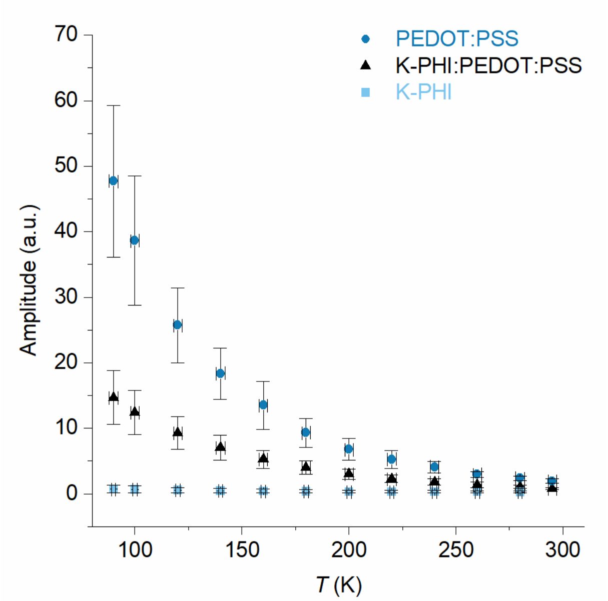

Figure S27. Amplitude of the EPR signal of K-PHI, PEDOT:PSS and K-PHI:PEDOT:PSS acquired at variable temperature.

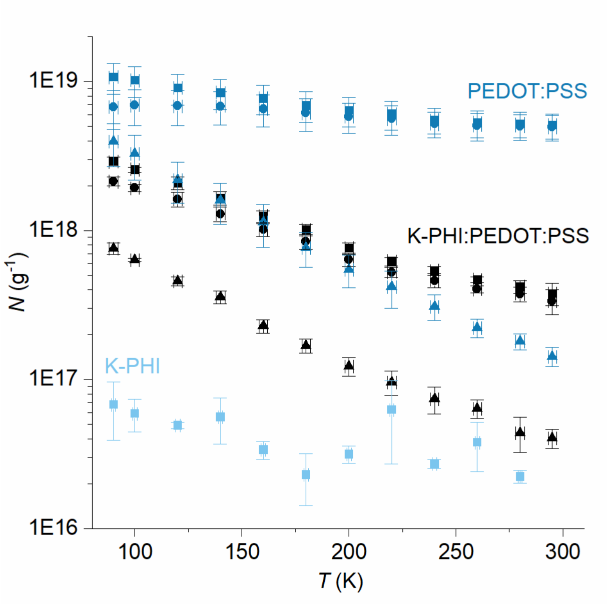

Figure S28. Total specific number of polarons (squares), specific number of polarons associated with the narrow component (triangles) and broad component (circles) in K-PHI, K-PHI:PEDOT:PSS and PEDOT:PSS. Higher standard deviation for K-PHI sample compared to PEDOT:PSS and K-PHI:PEDOT:PSS is explained by the relatively low concentration of radical species in the material and the low signal-to-noise ratio.

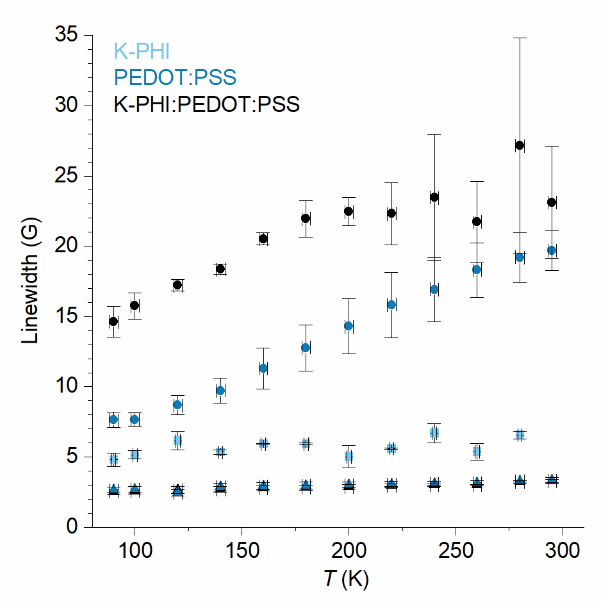

Figure S29. Linewidths of K-PHI (squares), narrow (triangles) and broad (circles) components of PEDOT:PSS and K-PHI:PEDOT:PSS derived from the fitting curves of the EPR spectra.

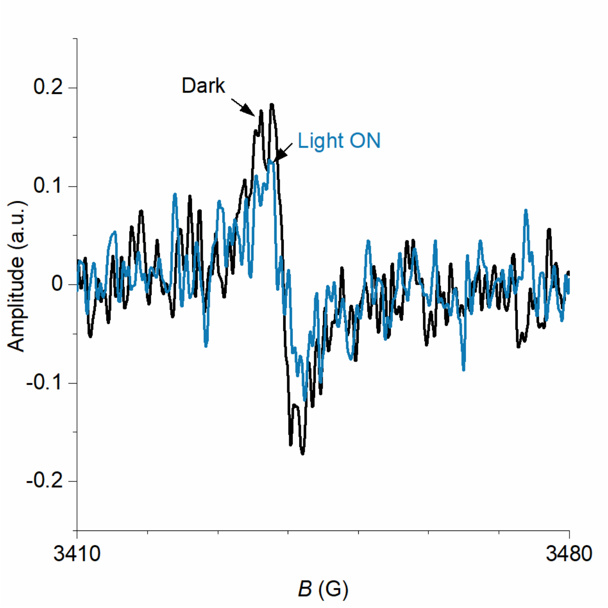

Figure S30. EPR spectra of K-PHI powder in dark and immediately after light irradiation.

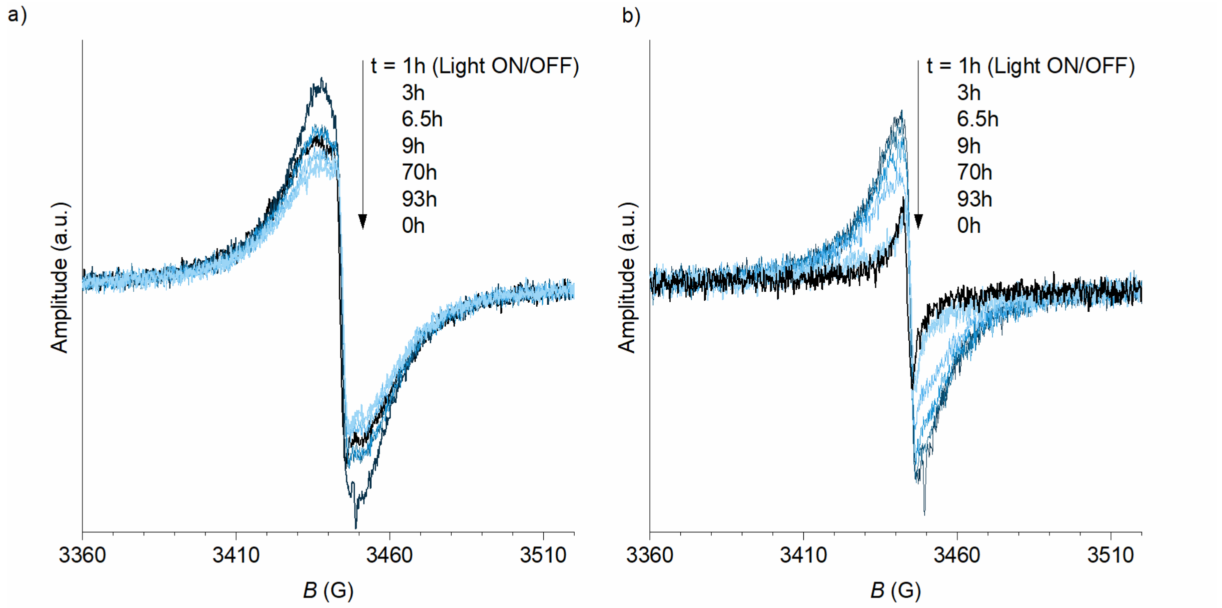

Figure S31. EPR spectra of PEDOT:PSS (a) and K-PHI:PEDOT:PSS (b) acquired in dark and after relaxation in the dark for a given period of time after irradiation with blue LED. Legends show the order of amplitude decrease in the acquired spectra.

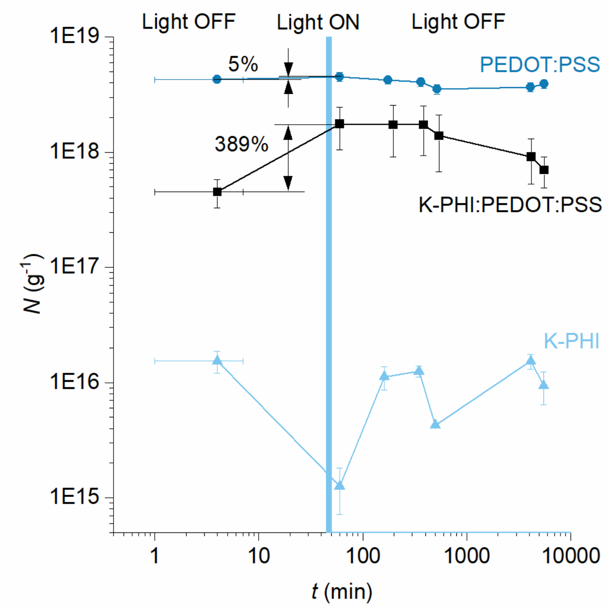

Figure S32. Time-dependent total specific concentration of radical species in K-PHI, and polarons in PEDOT:PSS and K-PHI:PEDOT:PSS determined from EPR spectroscopy. The non-monotonous behaviour for K-PHI is explained by the relatively low concentration of radical species in the material and the low signal-to-noise ratio.

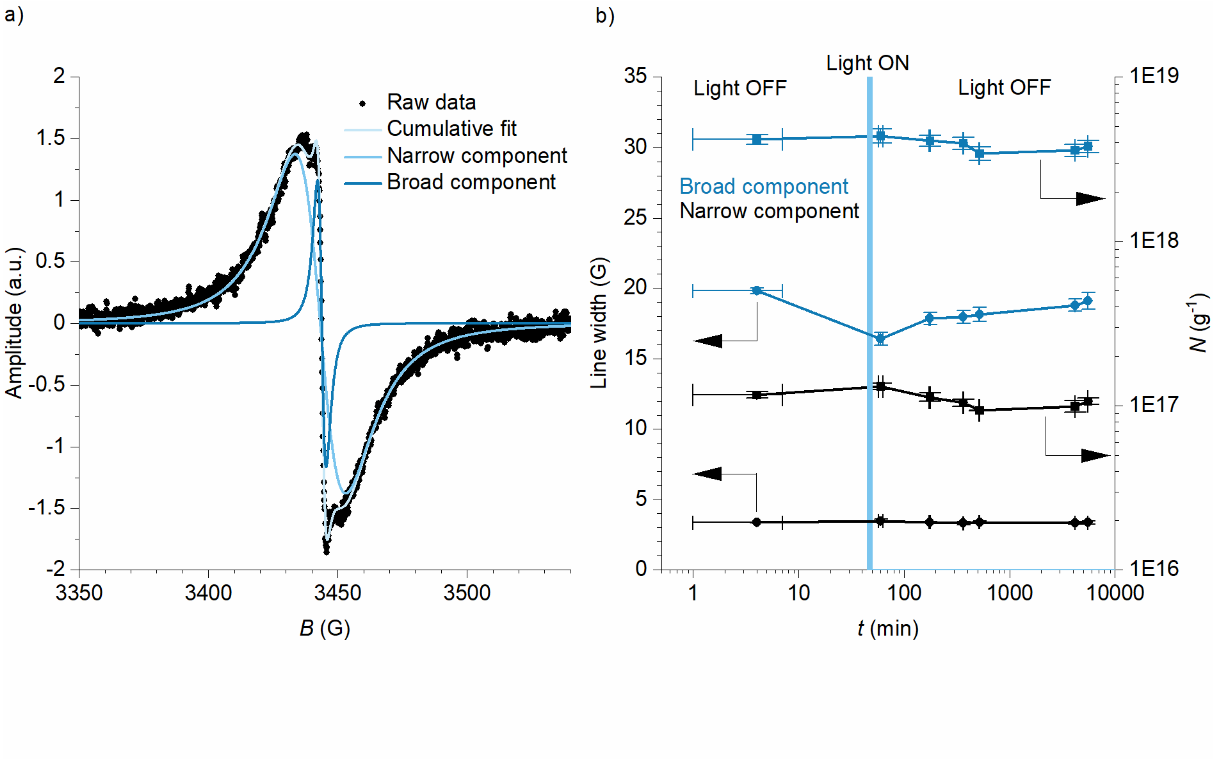

Figure S33. Time-dependent evolution of PEDOT:PSS EPR spectral parameters. a) An example of EPR spectrum of PEDOT:PSS acquired in the dark; b) Evolution of specific concentration of polarons associated with narrow and broad components and line width of PEDOT:PSS in the dark, upon irradiation with light for 5 min and the subsequent relaxation in the dark.

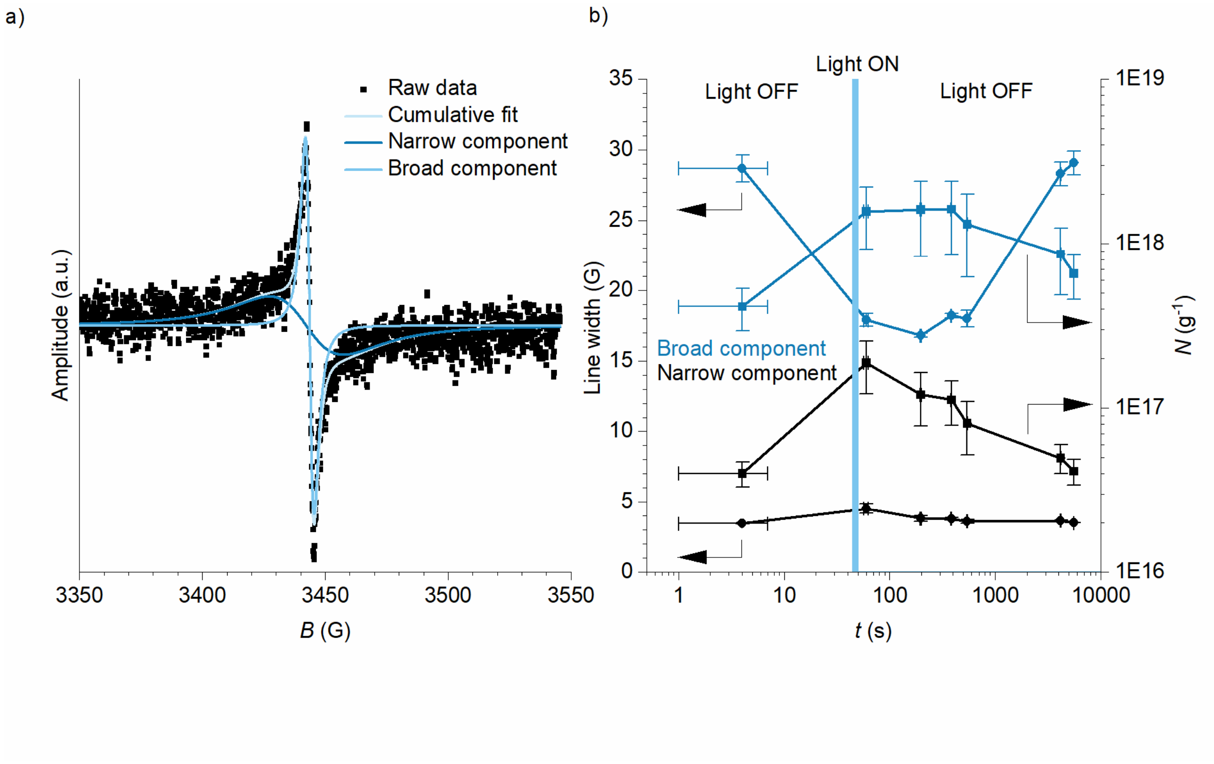

Figure S34. Deconvolution of K-PHI:PEDOT:PSS EPR spectrum. a) EPR spectrum acquired in dark, as an example; b) Evolution of specific concentration of polarons associated with narrow and broad components and line width upon K-PHI:PEDOT:PSS irradiation with light and the subsequent relaxation in dark for a specified period of time.

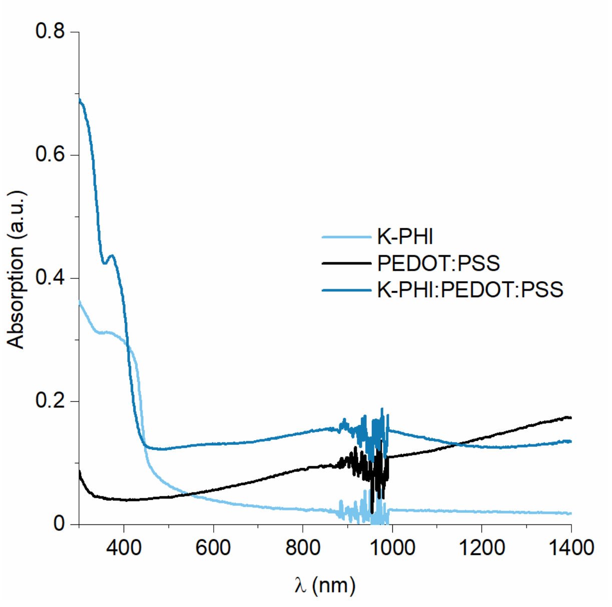

Figure S35. Steady-state absorption spectra of K-PHI, PEDOT:PSS and K-PHI:PEDOT:PSS films spray coated at FTO electrode.

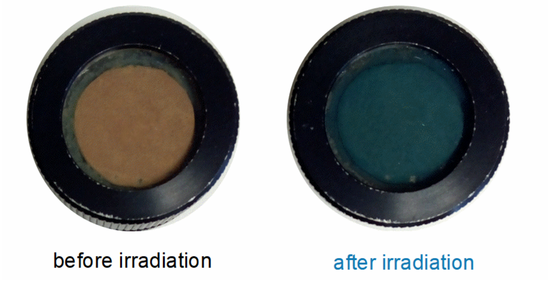

Figure S36. Suspension of K-PHI in benzylamine prior irradiation with light and immediately after irradiation with light. Blue colour indicates formation of long-lived radical of K-PHI.

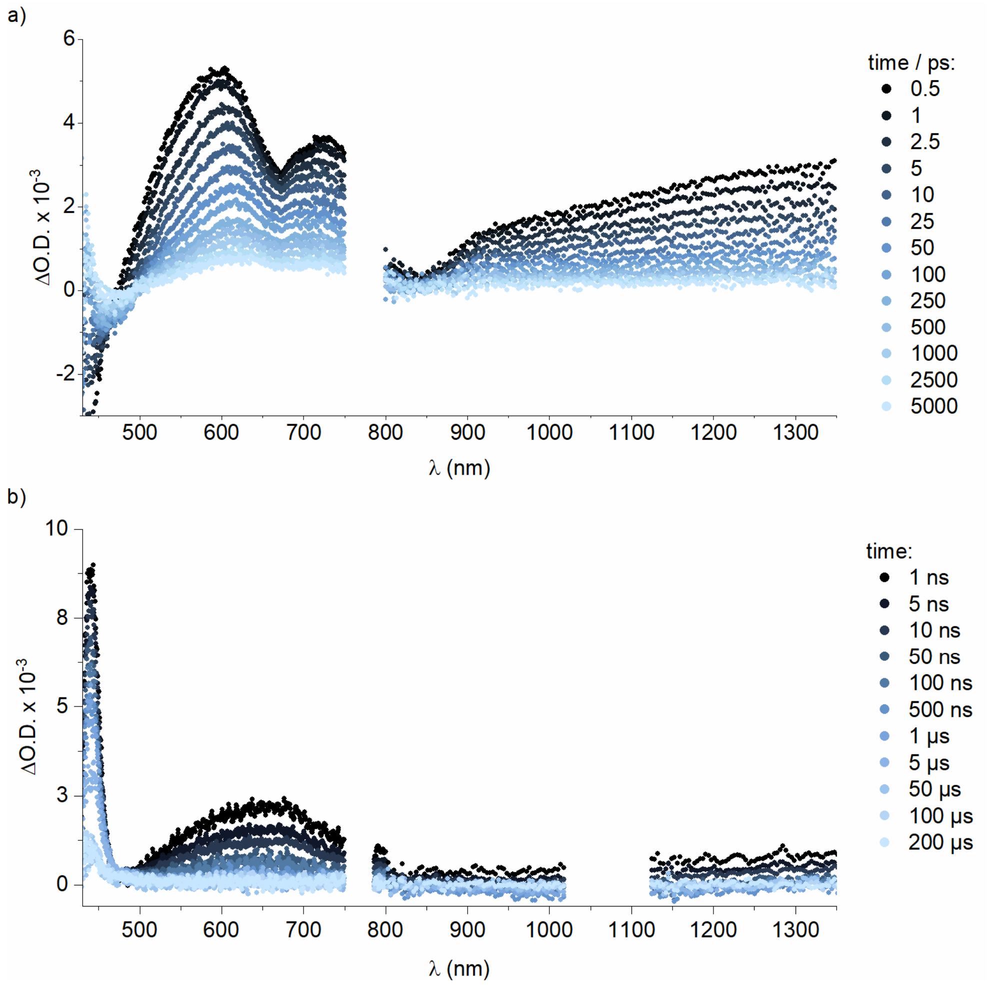

Figure S37. Differential absorption changes in the visible and near-infrared (nIR) region of the electromagnetic spectrum obtained upon a) sub-picosecond and b) nanosecond pump-probe transient absorption experiments (387 nm / 2 μJ) of K-PHI film on glass slide. The legend shows the respective time delays. Areas around 775 and 1064 nm are removed due to major contribution from the fundamental wavelength of the respective laser source used for white light generation or detector changes.

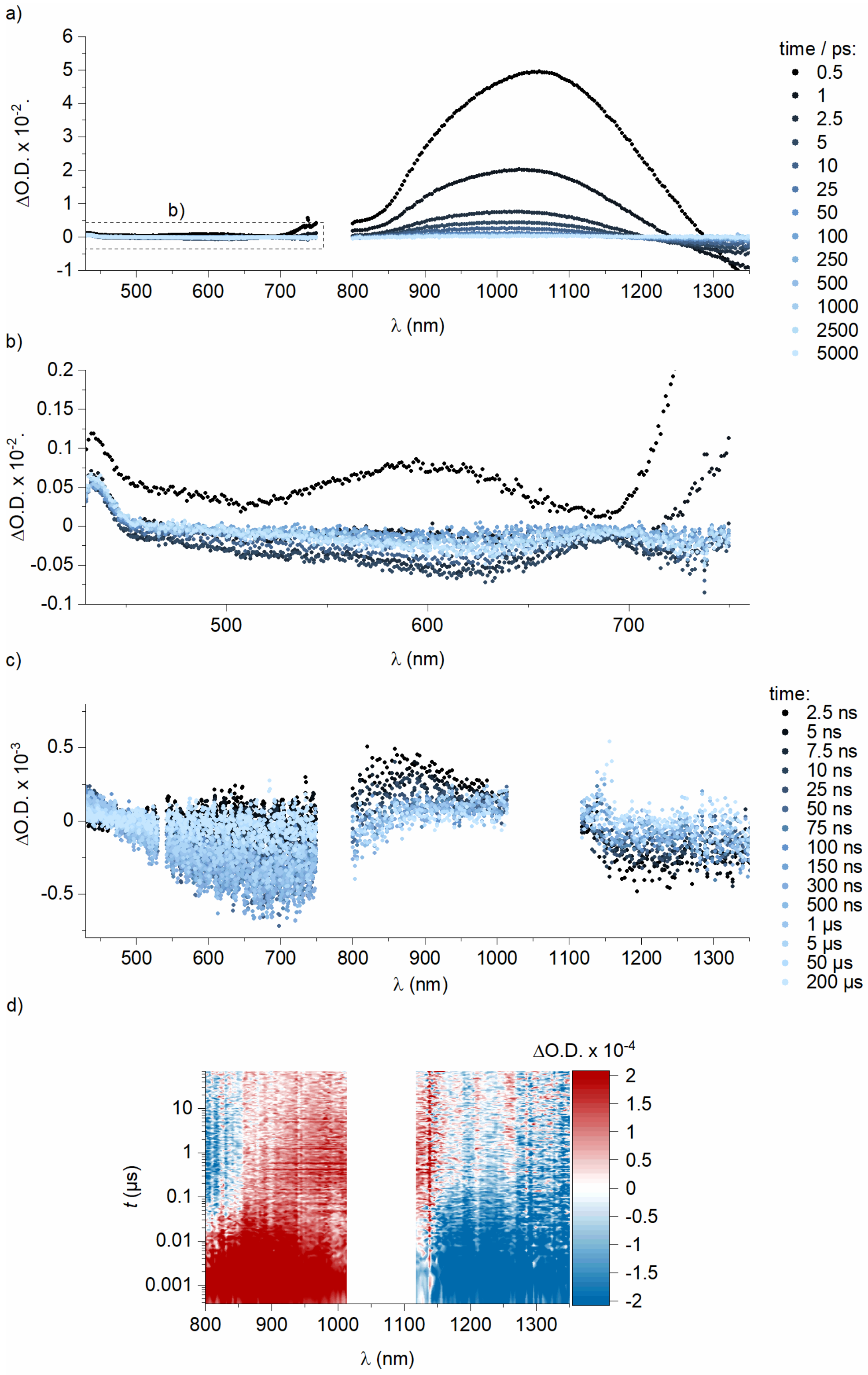

Figure S38. Differential absorption changes in the visible and near-infrared (nIR) region of the optical spectrum obtained upon a) sub-picosecond and c) nanosecond pump-probe transient

absorption experiments (387 nm / 2 µJ) of PEDOT:PSS film on glass slide. b) zoomed in version of the region between 430 and 750 nm. The legend shows the respective time delays. Areas around 775 and 1064 nm are removed due to major contribution from the fundamental wavelength of the respective laser source used for white light generation or detector changes. d) nsTA heat map of the differential absorption changes of the nIR optical spectrum of PEDOT:PSS slides on glass for better visibility of 1050 nm centered species.

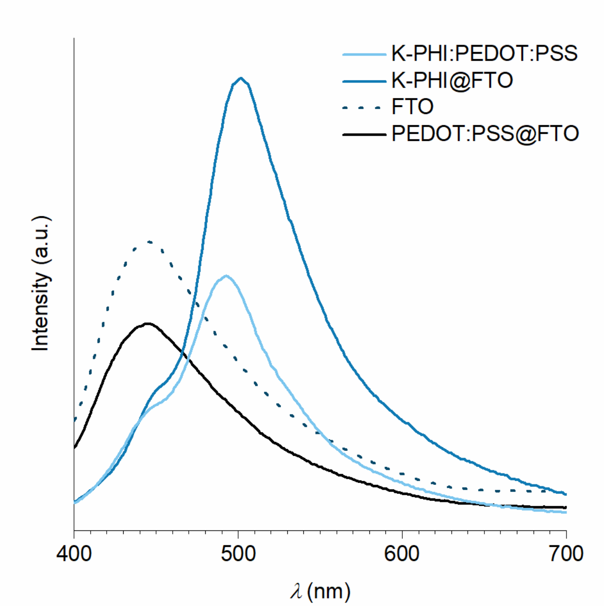

Figure S39. Photoluminescence spectra of the materials on FTO glass after photoexcitation at 360 nm.

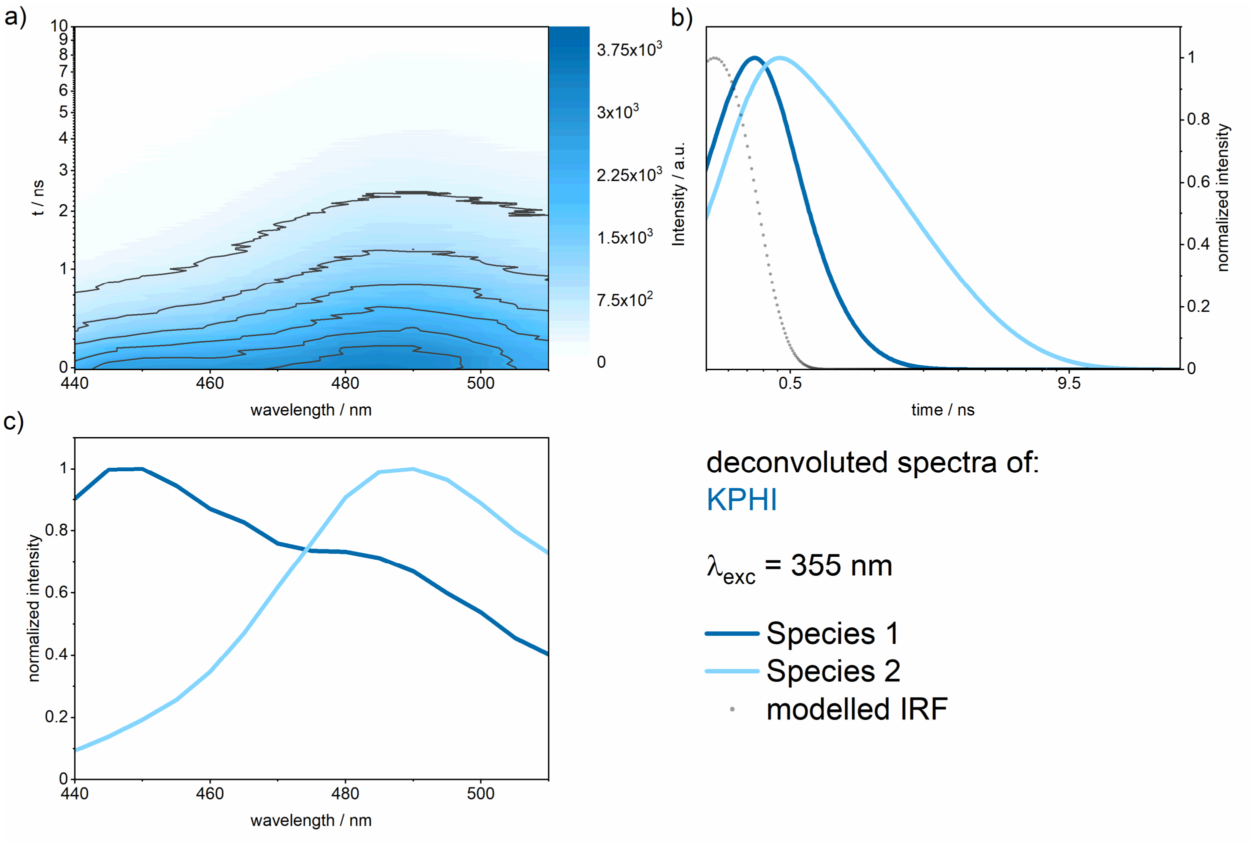

Figure S40. Time-resolved emission spectroscopy (TRES) of K-PHI film on glass slide obtained upon photoexcitation at $\lambda_{exc} = 355$ nm. a) Plot of time vs photoluminescence wavelength. b) Deconvolution of the TRES data with GloTarAn (global analysis) with two species, their individual time evolution and modelled instrument response function (IRF, block dots). c) The deconvolution of the TRES data of the two species taken into account for global analysis. Deconvoluted spectra of species 1 are in dark blue, species 2 in light blue.

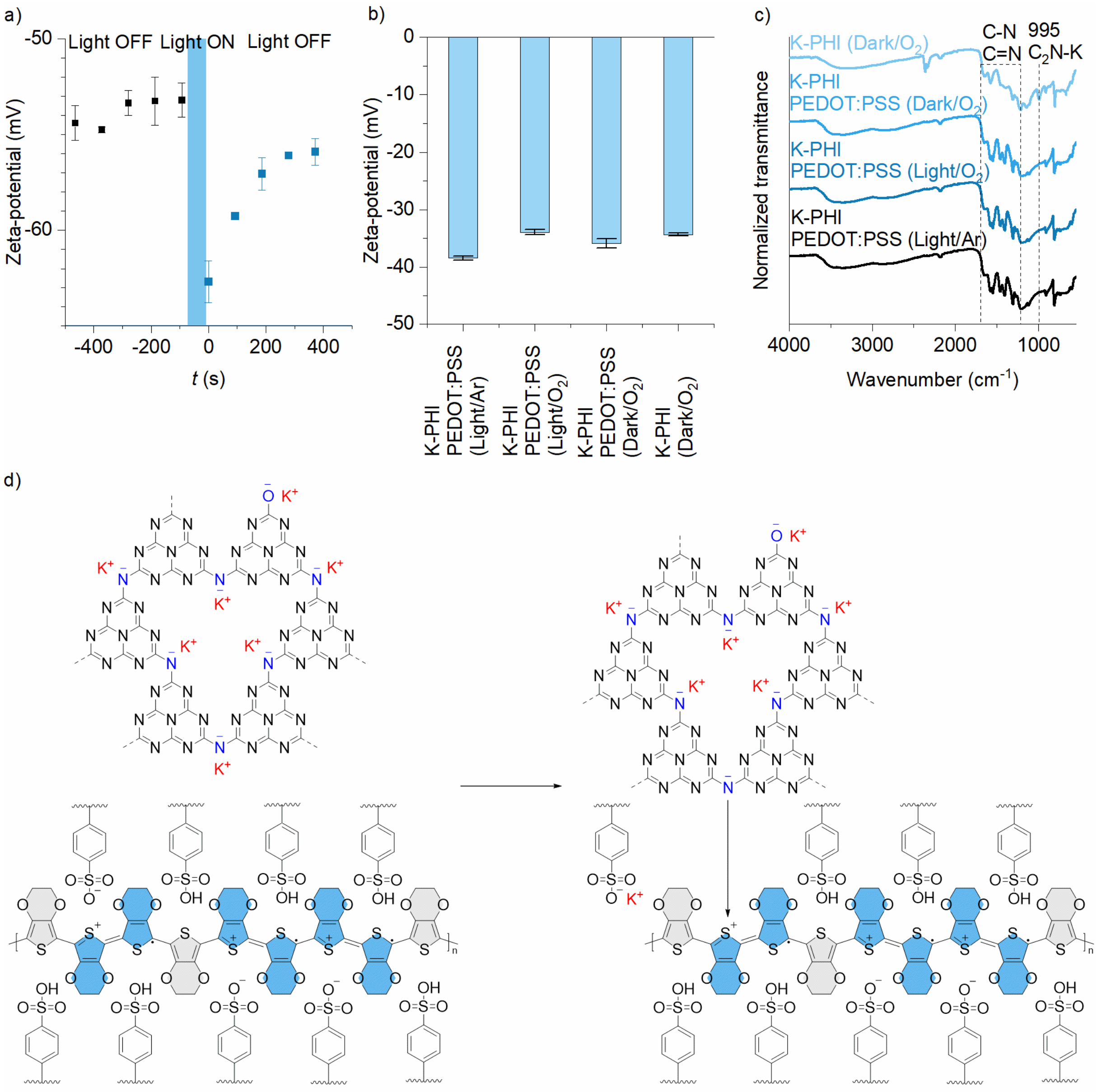

Figure S41. Mechanistic studies of K-PHI and (K-PHI) interactions with PEDOT:PSS. a) Zeta-potential of K-PHI particles in benzylamine:water (1:99 vol.) mixture in the dark and followed by relaxation in the dark after sample irradiation with blue light for 1 min; b) Zeta-potential of K-PHI particles recovered after stirring with PEDOT:PSS under light irradiation/in the dark under Ar/O_2; c) FT-IR spectra of K-PHI particles recovered after stirring with PEDOT:PSS under light irradiation/in the dark under Ar/O_2; d) Schematic mechanism of partial substitution of benzenesulfonic groups in PSS by K-PHI.

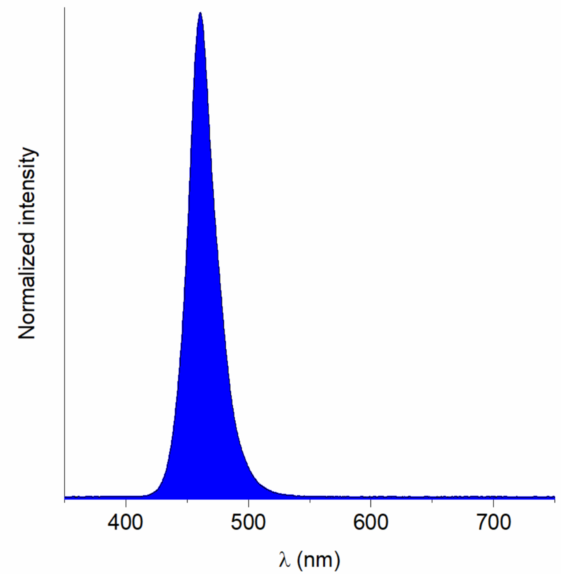

Figure S42. Emission spectrum of the LED used in the present work to study response of the hybrid nanocomposites to light.

8. Supplementary Tables

Table S1. PEDOT:PSS

Element	Content, wt. %
C	40.1±0.23
H	4.7±0.05
N	0.27±0.005
S	14.4±0.09

Table S2. K-PHI:PEDOT:PSS (28 wt. %)

Element	Content, %
C	36.6±0.05
H	3.9±0.1
N	13.8±0.3
S	10.2±0.2

Table S3. Semiconductor :PEDOT:PSS blends preparation

Entry	Semiconductor	ω,[a] wt. %	C_1,[b] mg mL^{-1}	V_1,[c] mL	V_2,[d] mL
1	Na-PHI	28	5	1	1
2	mpg-CN	28	5	1	1
3	g-CN	28	5	1	1
4	CdS	28	5	1	1
5	WO_3	28	5	1	1
6	TiO_2	28	5	1	1

[a] semiconductor content in the composite;

[b] semiconductor particles concentration in water;

[c] semiconductor particles suspension volume;

[d] PEDOT:PSS dispersion (1.3 wt. %) volume.

Table S4. Zeta-potentials of the materials[a]

Entry	Material	Zeta-potential±st.d., mV±mV
1	K-PHI	-33.5±0.2
2	PEDOT:PSS	-51.5±0.6

[a] measurements were performed in water

Table S5. Summary of roughness of PEDOT:PSS films prepared by different techniques and analyzed by AFM reported in literature.

Entry	Material	Technique	Field of view (µm x µm)	RMS Roughness, nm	Reference
1	K-PHI:PEDOT:PSS	Spray coating	10 x 10	44±8	This work
2	PEDOT:PSS	Spin-coating	1 x 1	1.58-1.76	[17]
3	PEDOT:PSS	Spin-coating	2 x 2	2.0±0.08	[18]
4	PEDOT:PSS	Spin-coating	~0.4 x 0.4	1.17	[19]
5	PEDOT:PSS	Solvent-casting	1 x 1	1.8	[20]
6	PEDOT:PSS	Solvent-casting	~0.5 x 0.5	1.6	[21]
7	PEDOT:PSS	Spin-coating	2.5 x 2.5	3.0-4.8	[22]
8	PEDOT:PSS	Spin-coating	20 x 20	0.9	[23]
9	PEDOT:PSS	Spin-coating	5 x 5	2.83	[24]
10	PEDOT:PSS	Spin-coating	2.5 x 2.5	1.49	[25]
11	PEDOT:PSS	Spin-coating	1 x 1	1.4	[26]
12	PEDOT:PSS treated with DMSO	Spin-coating	5 x 5	5.10	[27]
13	PEDOT:PSS	Spin-casting	0.4 x 0.4	8.622	[28]
14	PEDOT:PSS	Spin coating	8.7 x 8.7	0.99	[29]
15	PEDOT:PSS	Spin coating	2 x 2	1.01	[30]
16	PEDOT:PSS	Ink-jet printing	1 x 1	2.9	[31]
17	PEDOT:PSS	Substrate vibration-assisted spray coating	~4 x 4	8-49[a]	[32]
18	PEDOT:PSS	Spray coating	10 x 10	~11±2	[33]
19	PEDOT:PSS	Substrate vibration-assisted spray coating	10 x 10	3.3	[34]

[a] depends on the used conditions

9. References

1. Snellenburg, J. J.; Laptenok, S.; Seger, R.; Mullen, K. M.; van Stokkum, I. H. M., Glotaran: A Java-Based Graphical User Interface for the R Package Timp. *Journal of Statistical Software; Vol 1, Issue 3 (2012)* 2012.
2. Savateev, A.; Dontsova, D.; Kurpil, B.; Antonietti, M., Highly Crystalline Poly(Heptazine Imides) by Mechanochemical Synthesis for Photooxidation of Various Organic Substrates Using an Intriguing Electron Acceptor – Elemental Sulfur. *Journal of Catalysis* 2017, *350*, 203-211.
3. Savateev, A.; Kurpil, B.; Mishchenko, A.; Zhang, G.; Antonietti, M., A “Waiting” Carbon Nitride Radical Anion: A Charge Storage Material and Key Intermediate in Direct C–H Thiolation of Methylarenes Using Elemental Sulfur as the “S”-Source. *Chemical Science* 2018, *9*, 3584-3591.
4. Kurpil, B.; Otte, K.; Mishchenko, A.; Lamagni, P.; Lipiński, W.; Lock, N.; Antonietti, M.; Savateev, A., Carbon Nitride Photocatalyzes Regioselective Aminium Radical Addition to the Carbonyl Bond and Yields N-Fused Pyrroles. *Nature Communications* 2019, *10*, 945.
5. Ghosh, I.; Khamrai, J.; Savateev, A.; Shlapakov, N.; Antonietti, M.; König, B., Organic Semiconductor Photocatalyst Can Bifunctionalize Arenes and Heteroarenes. *Science* 2019, *365*, 360-366.
6. Chen, Z.; Savateev, A.; Pronkin, S.; Papaefthimiou, V.; Wolff, C.; Willinger, M. G.; Willinger, E.; Neher, D.; Antonietti, M.; Dontsova, D., “The Easier the Better” Preparation of Efficient Photocatalysts—Metastable Poly(Heptazine Imide) Salts. *Advanced Materials* 2017, *29*, 1700555.
7. Zabihi, F.; Xie, Y.; Gao, S.; Eslamian, M., Morphology, Conductivity, and Wetting Characteristics of Pedot:Pss Thin Films Deposited by Spin and Spray Coating. *Applied Surface Science* 2015, *338*, 163-177.
8. Huang, L.; Eedugurala, N.; Benasco, A.; Zhang, S.; Mayer, K. S.; Adams, D. J.; Fowler, B.; Lockart, M. M.; Saghayezhian, M.; Tahir, H.; King, E. R.; Morgan, S.; Bowman, M. K.; Gu, X.; Azoulay, J. D., Open-Shell Donor–Acceptor Conjugated Polymers with High Electrical Conductivity. *Advanced Functional Materials* 2020, *30*, 1909805.
9. London, A. E.; Chen, H.; Sabuj, M. A.; Tropp, J.; Saghayezhian, M.; Eedugurala, N.; Zhang, B. A.; Liu, Y.; Gu, X.; Wong, B. M.; Rai, N.; Bowman, M. K.; Azoulay, J. D., A High-Spin Ground-State Donor-Acceptor Conjugated Polymer. *Science Advances* 2019, *5*, eaav2336.
10. Lee, J.-K.; Cho, J. M.; Shin, W. S.; Moon, S. J.; Kemp, N. T.; Zhang, H.; Lamb, R., The Stability of Pedot:Pss Films Monitored by Electron Spin Resonance. *Journal of the Korean Physical Society* 2008, *52*, 621-626.
11. Lai, P. F.; Prawer, S.; Noble, C., Electron Spin Resonance Investigation of Ion-Irradiated Diamond. *Diamond and Related Materials* 2002, *11*, 1391-1396.
12. Zykwinska, A.; Domagala, W.; Czardybon, A.; Pilawa, B.; Lapkowski, M., In Situ Epr Spectroelectrochemical Studies of Paramagnetic Centres in Poly(3,4-Ethylenedioxythiophene) (Pedot) and Poly(3,4-Butylenedioxythiophene) (Pbudot) Films. *Chemical Physics* 2003, *292*, 31-45.
13. Mazzanti, S.; Kurpil, B.; Pieber, B.; Antonietti, M.; Savateev, A., Dichloromethylation of Enones by Carbon Nitride Photocatalysis. *Nature Communications* 2020, *11*, 1387.
14. Massonnet, N.; Carella, A.; Jaudouin, O.; Rannou, P.; Laval, G.; Celle, C.; Simonato, J.-P., Improvement of the Seebeck Coefficient of Pedot:Pss by Chemical Reduction Combined with a Novel Method for Its Transfer Using Free-Standing Thin Films. *Journal of Materials Chemistry C* 2014, *2*, 1278-1283.
15. Ghosh, S.; Inganäs, O., Self-Assembly of a Conducting Polymer Nanostructure by Physical Crosslinking: Applications to Conducting Blends and Modified Electrodes. *Synthetic Metals* 1999, *101*, 413-416.

16. Savateev, A.; Pronkin, S.; Willinger, M. G.; Antonietti, M.; Dontsova, D., Towards Organic Zeolites and Inclusion Catalysts: Heptazine Imide Salts Can Exchange Metal Cations in the Solid State. *Chemistry – An Asian Journal* 2017, *12*, 1517-1522.
17. Wang, X.; Kyaw, A. K. K.; Yin, C.; Wang, F.; Zhu, Q.; Tang, T.; Yee, P. I.; Xu, J., Enhancement of Thermoelectric Performance of Pedot:Pss Films by Post-Treatment with a Superacid. *RSC Advances* 2018, *8*, 18334-18340.
18. Gholampour, N.; Brian, D.; Eslamian, M., Tailoring Characteristics of Pedot:Pss Coated on Glass and Plastics by Ultrasonic Substrate Vibration Post Treatment. *Coatings* 2018, *8*, 337.
19. N'Konou, K.; Many, V.; Ruiz, C. M.; Treguer-Delapierre, M.; Torchio, P., Effect of Shell Thickness of Gold-Silica Core-Shell Nanospheres Embedded in an Organic Buffer Matrix for Plasmonic Solar Cells. *Journal of Applied Physics* 2018, *123*, 063102.
20. Zhu, Q.; Yildirim, E.; Wang, X.; Soo, X. Y. D.; Zheng, Y.; Tan, T. L.; Wu, G.; Yang, S.-W.; Xu, J., Improved Alignment of Pedot:Pss Induced by in-Situ Crystallization of "Green" Dimethylsulfone Molecules to Enhance the Polymer Thermoelectric Performance. *Frontiers in Chemistry* 2019, *7*.
21. Lu, B.; Yuk, H.; Lin, S.; Jian, N.; Qu, K.; Xu, J.; Zhao, X., Pure Pedot:Pss Hydrogels. *Nature Communications* 2019, *10*, 1043.
22. D'Angelo, P.; Tarabella, G.; Romeo, A.; Marasso, S.; Verna, A.; Cocuzza, M.; Peruzzi, C.; Vurro, D.; Iannotta, S., Pedot:Pss Morphostructure and Ion-to-Electron Transduction and Amplification Mechanisms in Organic Electrochemical Transistors. *Materials* 2018, *12*.
23. Du, X.; Lytken, O.; Killian, M. S.; Cao, J.; Stubhan, T.; Turbiez, M.; Schmuki, P.; Steinrück, H.-P.; Ding, L.; Fink, R. H.; Li, N.; Brabec, C. J., Overcoming Interfacial Losses in Solution-Processed Organic Multi-Junction Solar Cells. *Advanced Energy Materials* 2017, *7*, 1601959.
24. Jian-Feng, L.; Chuang, Z.; Heng, Z.; Jun-Feng, T.; Peng, Z.; Chun-Yan, Y.; Yang-Jun, X.; Duo-Wang, F., Improving the Performance of Perovskite Solar Cells with Glycerol-Doped Pedot:Pss Buffer Layer. *Chinese Physics B* 2016, *25*, 028402.
25. Gasiorowski, J.; Menon, R.; Hingerl, K.; Dachev, M.; Sariciftci, N. S., Surface Morphology, Optical Properties and Conductivity Changes of Poly(3,4-Ethylenedioxythiophene):Poly(Styrenesulfonate) by Using Additives. *Thin Solid Films* 2013, *536*, 211-215.
26. Thomas, J. P.; Zhao, L.; McGillivray, D.; Leung, K. T., High-Efficiency Hybrid Solar Cells by Nanostructural Modification in Pedot:Pss with Co-Solvent Addition. *Journal of Materials Chemistry A* 2014, *2*, 2383-2389.
27. Jucius, D.; Lazauskas, A.; Grigaliknas, V.; Gudaitis, R.; Guobien , A.; Prosy evas, I.; Abakevi ien , B.; Andrulevi ius, M., Structure and Properties of Dual-Doped Pedot:Pss Multilayer Films. *Materials Research* 2019, *22*.
28. Rutledge, S. A.; Helmy, A. S., Etch-Free Patterning of Poly(3,4-Ethylenedioxythiophene)–Poly(Styrenesulfonate) for Optoelectronics. *ACS Applied Materials & Interfaces* 2015, *7*, 3940-3948.
29. Khodakarimi, S.; Hekhmatshoar, M. H.; Nasiri, M.; Khaleghi Moghaddam, M.; Abbasi, F., Effects of Process and Post-Process Treatments on the Electrical Conductivity of the Pedot:Pss Films. *Journal of Materials Science: Materials in Electronics* 2016, *27*, 1278-1285.
30. Kumar, P.; Kumar, A.; Shin, P.-K.; Ochiai, S., Influence of Solvent Treatment with Fluoro Compounds on the Properties of Poly(3,4-Ethylenedioxythiophene):Poly(Styrene Sulfonate) Polymer as a Hole Transport Layer in Polymer Solar Cells. *Journal of Photonics for Energy* 2014, *4*, 043097.
31. Soleimani-Gorgani, A., Co-Solvents Roles in Pedot:Pss Ink-Jet Inks. *Advances in Natural Sciences: Nanoscience and Nanotechnology* 2018, *9*, 025009.
32. Zabihi, F.; Eslamian, M., Substrate Vibration-Assisted Spray Coating (Svasc): Significant Improvement in Nano-Structure, Uniformity, and Conductivity of Pedot:Pss Thin Films for Organic Solar Cells. *Journal of Coatings Technology and Research* 2015, *12*, 711-719.

33. Xie, Y.; Gao, S.; Eslamian, M., Fundamental Study on the Effect of Spray Parameters on Characteristics of P3ht:Pcbm Active Layers Made by Spray Coating. *Coatings* 2015, *5*, 488-510.
34. Soltani-kordshuli, F.; Zabihi, F.; Eslamian, M., Graphene-Doped Pedot:Pss Nanocomposite Thin Films Fabricated by Conventional and Substrate Vibration-Assisted Spray Coating (Svasc). *Engineering Science and Technology, an International Journal* 2016, *19*, 1216-1223.
